# Comprehensive evaluation method of stope stability and its application in deep metal mine

**DOI:** 10.1371/journal.pone.0283205

**Published:** 2023-03-17

**Authors:** Zhaojun Qi, Huanxin Liu, Zhihai An, Yantian Yin, Zhen Liu, Mingde Zhu, Yunsen Wang, Hong Zhou

**Affiliations:** 1 Deep Mining Laboratory of Shandong Gold Group Co., Ltd., Jinan, China; 2 Key Laboratory of Ministry of Education on Safe Mining of Deep Metal Mines, Northeastern University, Shenyang, China; University of Science and Technology Beijing, CHINA

## Abstract

The problem of high stress conditions is a critical challenge for mining activity in deep metal mines, and the difficulty and economic cost of maintaining stope stability also increases. In order to ensure stope stability in the mining process, it is necessary to evaluate stope stability for a more stable stope structure. However, there are many kinds of methods for rock stability assessment, and most of them are based on empirical and analytical methods, which lead to uncertain results. In this paper, a comprehensive evaluation method including factors of stope stability, self-stabilization time, maximum safety span, and support measure is determined, which is more likely to provide a comprehensive result. Finally, it was verified by the field application in Jiaojia Gold Mine. Results show that it can accurately evaluate the stope stability from different aspects compared with the traditional single evaluation method, and it leads to a more comprehensive and reliable result.

## 1 Introduction

The mining industry supports the sustainable development of the national economy. With the reduction and depletion of shallow mineral resources, it has become an inevitable trend in the world mining industry to utilize deep resources. More than 80 metal mines, mostly in South Africa, are over 1000 meters deep, such as TauTona Gold Mine in Witwatersrand (3910m), East Rand Gold Mine (3585m), Mponeng Gold Mine (4500m), Anglogold Gold Mine (3700) and the deepest platinum-palladium mine in South Africa (2200m). Moreover, there are 16 metal mines with a mining depth of over 1000m in China. In the future, with the development of exploration technology and equipment, it is entirely possible to find a large number of metal deposits in the depth of 3000~5000m.

With the mining activity going deeper, the ground stress increases, the mining technical conditions and environment deteriorate seriously, and the mining cost increases sharply. The premise of mining is to ensure the stability of rock mass during the mining activity, so it is necessary to design a reasonable stope structure parameter that could ensure its stability. From the actual production situation of the mining site, the structural parameters of the underground stope occupy a decisive role in its stability. Meanwhile, it affects the economic benefits of mining. When this parameter is too large, it will lead to instability and caving of surrounding rock, which also increases the dilution and risk of operation. When it goes too small, resulting in a large mining volume, low ore recovery rate, and economic efficiency [1, 2]. Therefore, stope stability is an important index to evaluate the rationality of stope structure parameters. Stope instability directly affects ore loss and dilution, reduces mine economic benefits, and even affects mine safety production.

Many scholars have done lots of research on stope stability. Mathews et al. first proposed the stability diagram method in 1981 [3], which is an empirical formula summarizing from a large number of engineering examples. In 1988, Potvin revised Mathews stability chart by analyzing 242 cases and redefining some adjustment factors [4]. In 1992, Potvin and Nickson et al. verified the rationality of this method and proposed amendments by collecting more deep mining field data [5]. In 2000, Trueman et al. used the method of logarithmic regression to redefine the stable area and serious damage area based on a large number of newly added case data [6]. Moreover, Mawdeskey et al. gave determined the equal probability diagram of these two areas in 2001 [7]. The general step of using Mathews stability diagram method to evaluate the stope is to calculate the stability number N and hydraulic radius S on the basis of numerical simulation, draw those parameters on the diagram, and evaluate stope stability according to the diagram area where the intersection is located.

With the revolution of computer technology, the application of the numerical analysis method of rock mass engineering has become more extensive and necessary for studying the stability of underground rock mass. Elrawy Abdellah et al. [8] used RS^2D^ to study the depth of stope failure and evaluated the stope stability according to the failure depth of the plastic zone, the range of yield or plastic zone and the deformation profile.

Aiming at the problem that the goaf area is hazardous, Wu et al. [9] established the limit equation of pillar under steady state and introduced interval theory by using the Hoek-Brown strength criterion. This method analyzes the failure degree of rock mass through the weight of overlying strata, uniaxial compressive strength of rock, and failure degree of rock, and then evaluates the stability of goaf.

With the assistance of acoustic, optical, or electrical sensors and equipment, the acoustic signals of surrounding rock displacement, stress, and rock fracture are collected and analyzed to evaluate the stability of the stope. At present, the main technology of goaf stability monitoring in China are the micro-seismic monitoring method, photo-elastic stress meter method, acoustic emission monitoring method, three-dimensional laser scanning method, etc. Zhao Kui et al. [10] used a long-term photo-elastic stress meter to monitor the stress change in the mining activity in the process of studying the stability of goaf in a gold mine, and carried out cluster analysis to classify goaf stability into three levels and identify pillars that play a key role in goaf stability.

However, there is no unified evaluation method for stope stability. According to the common method in engineering, a comprehensive way is proposed in this paper. Firstly, the geomechanical data is investigated, and then the RMR and Q values are calculated. Survey and calculation results are input into the database. RMR, Q value and other information of the field point are calculated by inverse distance weighted average method, Chinese national standard recommendation method, self-stabilization time judgment method, and Barton stope width empirical formula are used to evaluate the stability of stope. At the same time, the support parameters are calculated by using the support recommendation formula, Chinese national standard recommendation method, and Q grading support chart theory. These stability evaluation results and support parameters are compiled into a table. Through the mutual confirmation of each result, a multidimensional comprehensive evaluation system is formed from stope stability, self-stabilization time, safety span and support measures. In order to be quickly applied on site, the stability evaluation system was developed by python and applied in Sizhuang mining area of the Jiaojia gold mine. Results show that the method and system provided in this paper can quickly evaluate the stability of the designed stope, and the evaluation results are basically consistent with the actual monitoring results.

## 2 Methodology

**Fig 1:**
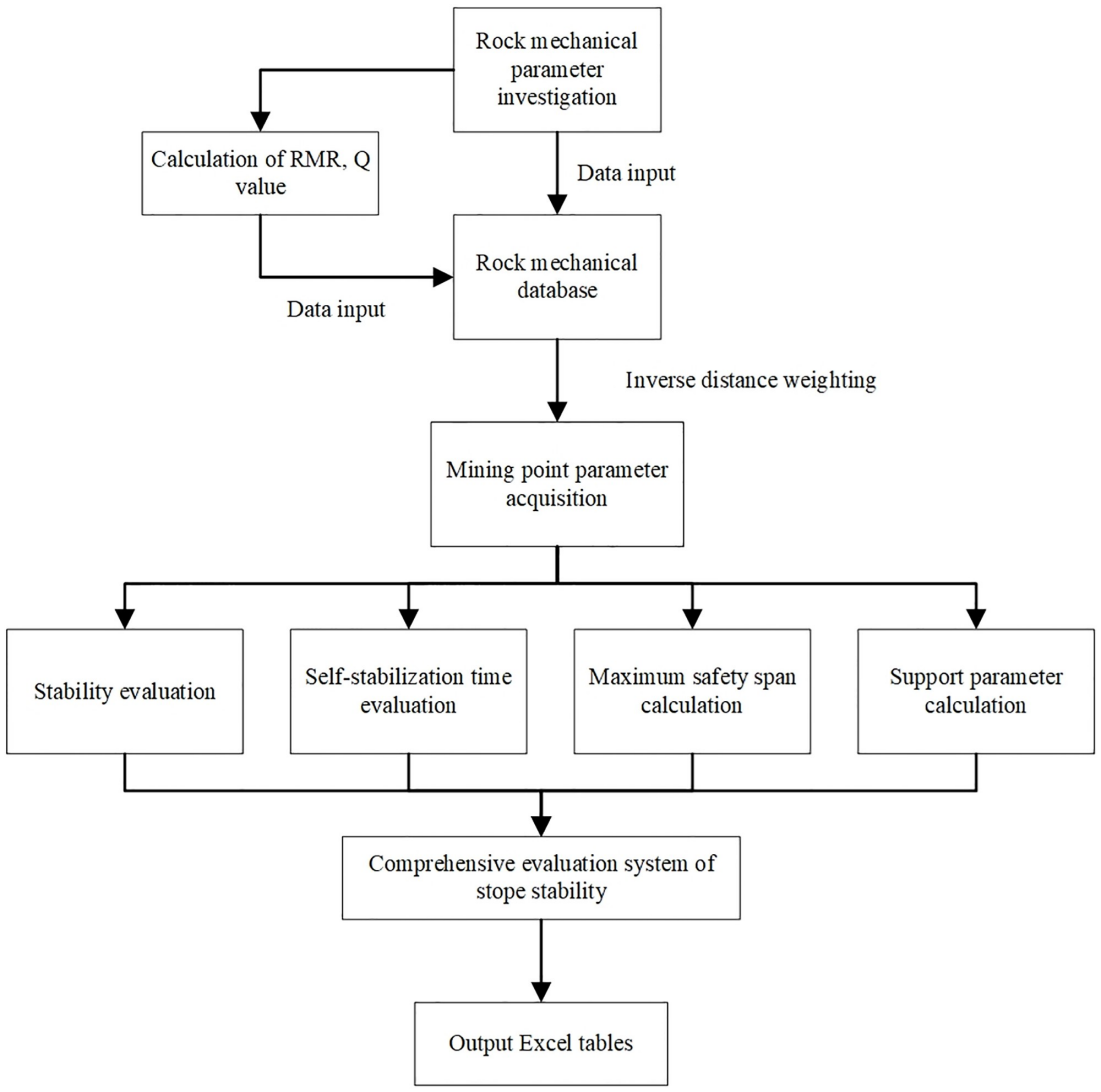
Flow chart of comprehensive evaluation method.

Through the investigation of rock mechanics parameters on site, using the results of rock mass quality classification, combined with Mathews stability chart, limit span method for span, Chinese national standard recommendation method, self-stability time judgment method, and other methods, the comprehensive evaluation of stope stability is carried out. At the same time, the evaluation results are output into an Excel sheet. This paper proposes a specific flow chart of the comprehensive evaluation method as shown in Fig 1.

### 2.1 RMR and Q value calculation

Rock mass quality evaluation and classification are essential work to evaluate the stability of rock mass. Whereas the RMR and Q system are more familiar, most stability evaluation methods use index values of these two classification methods, so it is necessary to measure.

Rock mass rating is proposed by Bieniawski from CSRI of South Africa [11, 12]. It consists of five indexes: rock fragmentation, RQD, joint spacing, joint condition, and groundwater. Each value is scored according to the established criteria, and the RMR can be obtained by adding these values.


$$RMR=R_{1}+R_{2}+R_{3}+R_{4}+R_{5}+R_{6}$$ (1)

In the formula: R_1_ is the rock mass strength (point load strength or uniaxial compressive strength); R_2_ stands for the core quality index RQD value; R_3_ represents the joint spacing score value; R_4_ is the joint condition score value; R_5_ is the groundwater condition value; R_6_ is the modified parameter scoring system.

The Q system classification method was proposed by Barton of the Norwegian Institute of Geotechnical Engineering in 1974 [13]. It is calculated by six indexes including rock mass quality index, joint group number, roughness system, joint alteration coefficient, fissure water reduction coefficient, and in-situ stress conditions. Similar to the calculation of RMR, the above six indexes are scored, and then the score value is substituted into the following formula to calculate the Q value [14]:

$$Q=\frac{RQD}{J_{n}}\cdot \frac{J_{r}}{J_{a}}\cdot \frac{J_{\omega }}{SFR}$$ (2)

Where *RQD* is the rock mass quality index; *J*_*n*_ is the number of joint groups; *J*_*r*_ is the roughness system; *J*_*a*_ is the joint alteration coefficient; *J*_*w*_ is the fracture water reduction coefficient; *SRF* is the stress reduction coefficient.

### 2.2 Inverse distance weight method

The investigation of rock mechanics parameters is a sampling survey in the stope or development roadway. These survey data generally reflect the surrounding rock state of the corresponding mining area, and cannot be specific to each point. The inverse distance weight could be utilized to identify the mechanical parameter at a specific point.

Inverse Distance Weight (IDW) is a spatially weighted average interpolation method that can be exact or smooth interpolation [15]. It is used to calculate the attribute value of the unsampled point, assuming that the value is a weighted average of the known values in the neighborhood [16]. The calculation method is as follows:

$$P_{i}=\sum_{j=1}^{n}\lambda_{j}P_{j}$$ (3)

$$\lambda_{i}=\;\frac{d_{ij}{}^{-\alpha }}{\sum_{j=1}^{n}d_{ij}{}^{-\alpha }}$$ (4)

$$\sum_{j=1}^{n}\lambda_{j}=1$$ (5)

Where *P*_*i*_ is the parameter values of points to be solved; *P*_*j*_ is the parameter values of known points in the region; *λ*_*i*_ is the weight of known Points in Region; *n* is number of known points in a region; *d*_*ij*_ is the distance from the point i to the point j; *α* is weight power function.

### 2.3 Stope stability calculation

#### 2.3.1 Mathew stability chart method

The Mathews stability diagram method proposed by Mathews et al. [17] is based on the calculation of stability coefficient N and hydraulic radius S, and combines the stability diagram to evaluate the stability of underground stope under supporting and non-supporting conditions. Then Potvin(4) made improvements, and the expression of the stability coefficient is as follows:

$$N=\frac{RQD}{J_{n}}\cdot \frac{J_{r}}{J_{a}}\cdot A\cdot B\cdot C$$ (6)

Where N is the stability coefficient; A is the rock stress coefficient; b is the correction coefficient of joint orientation; c is the gravity adjustment coefficient of the designed exposed face; and other symbols are the same as above.

**Fig 2:**
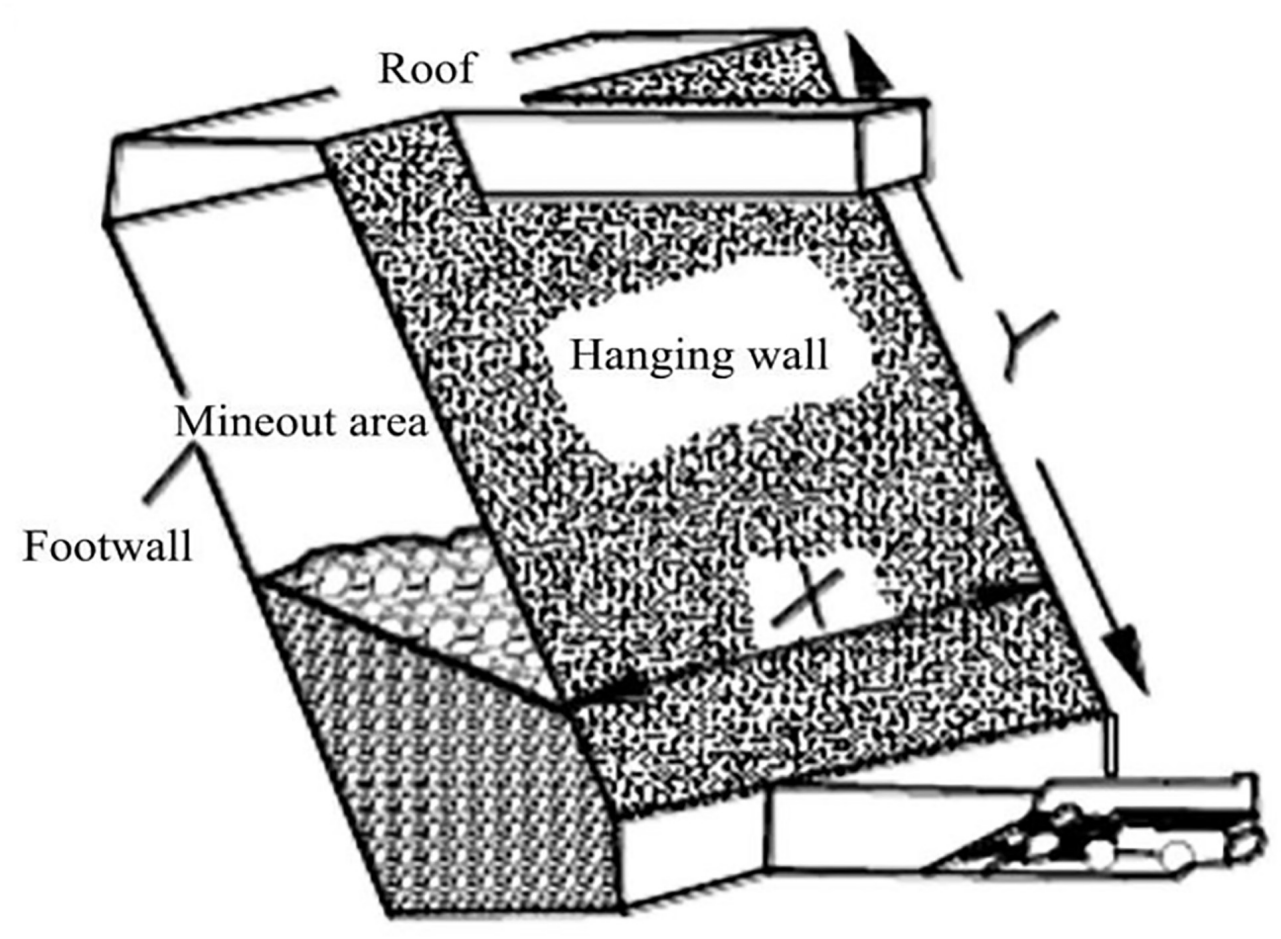
Hydraulic radius determination method diagram.

Hydraulic radius HR reflects the size and shape of the stope. The hydraulic radius is the ratio of the excavation surface to the perimeter of the exposed surface, which can be determined by the following method shown in Fig 2, the hydraulic radius of the hanging wall is equal to the area divided by the perimeter.

The calculation method of hydraulic radius S is:

$$S=\frac{L\times H}{2\times \left(\text{L}+\text{H}\right)}$$ (7)

Where L and H are the length and width of the exposed surface respectively.

**Fig 3:**
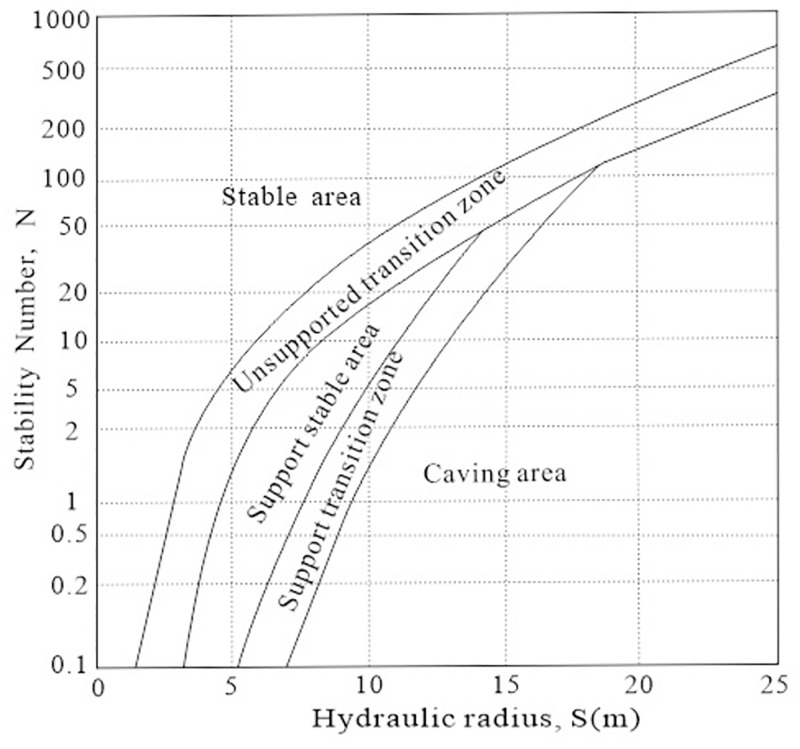
Mathews stability chart.

After obtaining the stability coefficient and hydraulic radius, the stability of the stope under the current structural parameters is judged according to Fig 3.

#### 2.3.2 Limit span method

**Fig 4:**
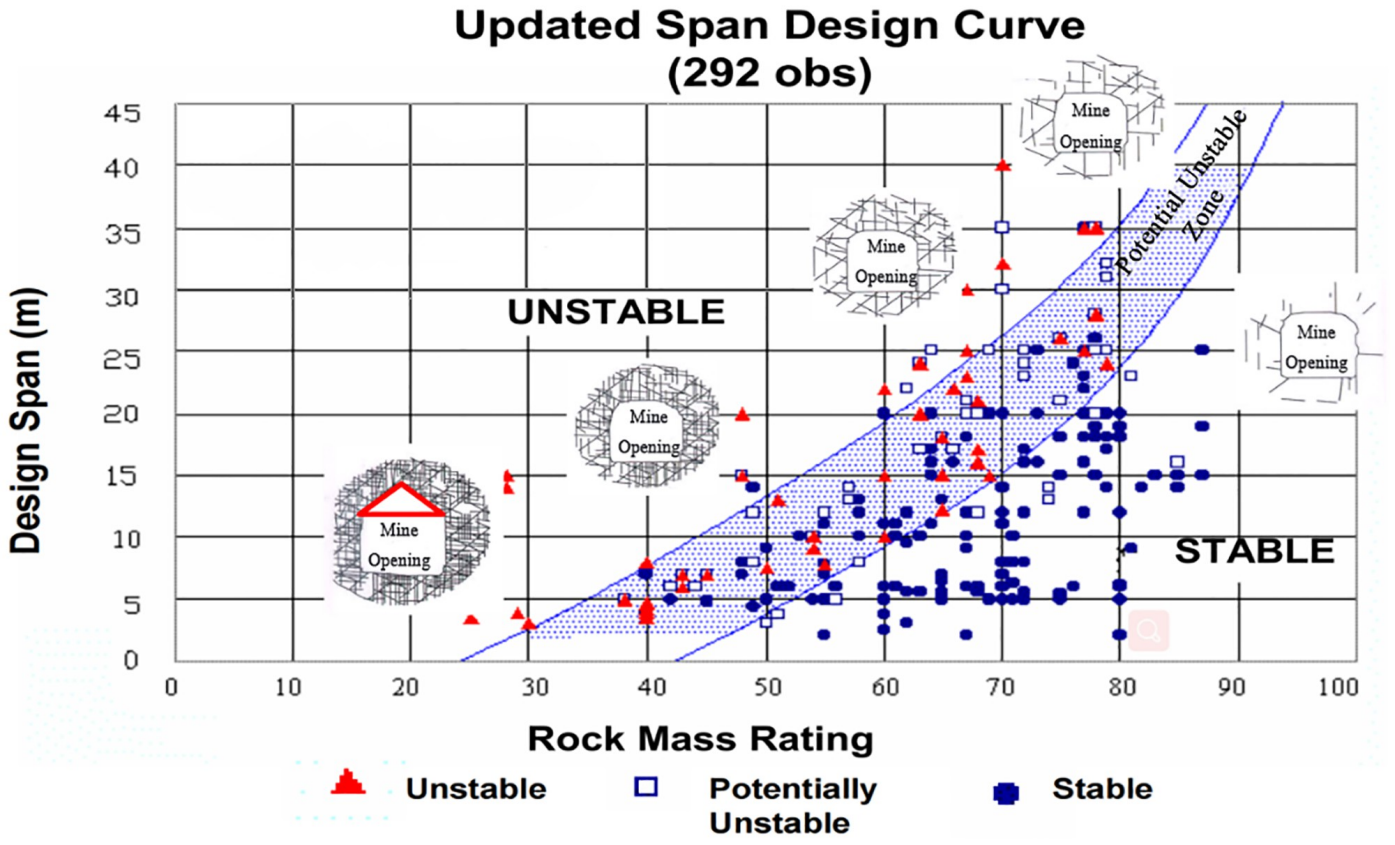
Limit span method (under the condition of no support or local support).

**Fig 5:**
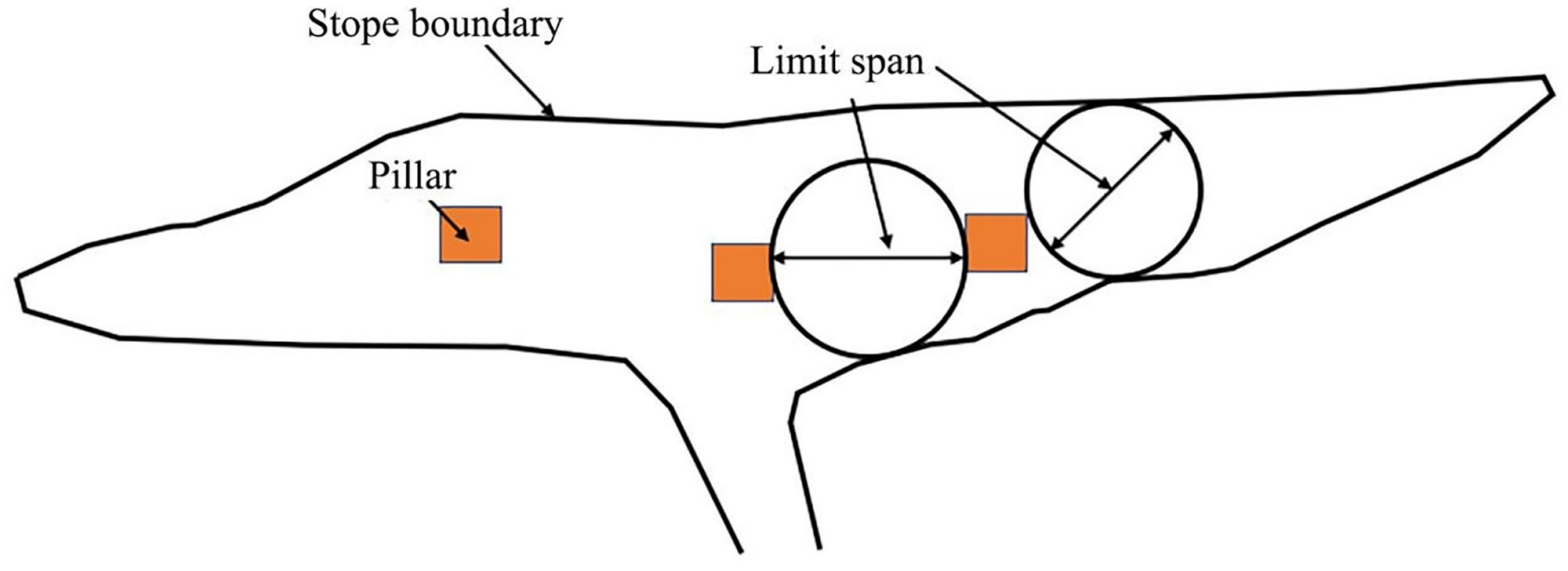
Determination of limit span of the stope.

The research team of UBC University in Canada obtained the relationship between rock mass quality RMR and the limit span of the stope by analyzing 292 examples of stope roof stability situations mined by different layered filling methods in Canada [18], and established the limit span curve, as shown in Fig 4 [19]. It can be seen from Fig 4 that the limit span curve divides the surveyed observation points into three regions: stable, potential unstable (fluctuation), and unstable. Based on this, the stope span can be designed, or the stability of the surrounding rock under a certain span can be evaluated. It should be noted that the limit span in this method is defined as the diameter of the maximum inscribed circle within the exposed roof boundary, and its determination method is shown in Fig 5.

In addition, since the limit span method is based on the RMR evaluation method, when there are obvious gently inclined joints (joint dip angle is less than 30 degrees), the RMR value in this area needs to be further reduced by 10.

### 2.4 Stope self-stabilization time calculation

#### 2.4.1 Self-stabilizing time evaluation (based on RMR classification)

**Fig 6:**
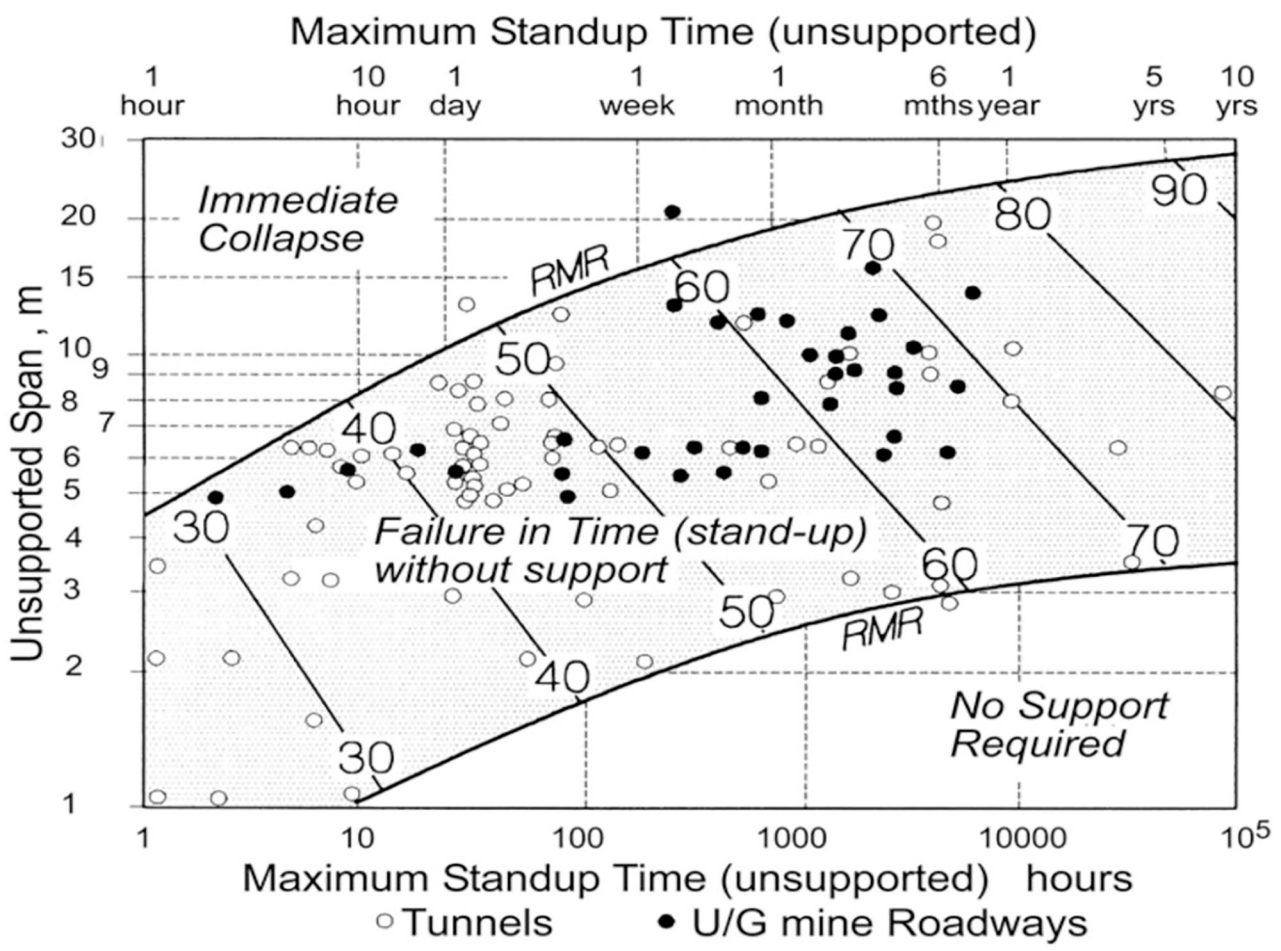
Self-stabilization time judgment based on RMR classification.

The stability time of surrounding rock is also an important index to evaluate stope stability. Its value can not only reflect the quality of surrounding rock in the stope, but also provide suggestions for selecting excavation and support method. In the study of self-stabilizing time, Bieniawski [20] summarized the relationship between the structural parameters of the unsupported stope (exposed surface span) and its self-stabilizing time in 1993 based on the RMR classification of rocks, as shown in Fig 6. And it can be seen from Fig 6 that the self-stabilizing time can be estimated according to the RMR value of the surrounding rock and the unsupported span, and the larger the RMR value and the smaller the span design, the longer the self-stabilizing time of the surrounding rock.

#### 2.4.2 Chinese national standard recommendation method

The above self-stabilizing time assessment method only obtains the self-stabilizing time according to RMR and span, which result is slightly rough. Based on this, China’s national standard “Technical Code for Rock and Soil Anchor and Shotcrete Support Engineering” (GB 50086–2015) [21] determined the provisions on the level of surrounding rock of tunnel caverns, which makes the evaluation to the stability of stope or tunnel without support from rock mass structure, rock strength, rock mass acoustic wave and rock mass stress ratio, and uses multiple indicators. Those results not only estimate the self-stabilization time but also give the failure form of the surrounding rock.

The rock mass integrity index K_v_ can be calculated as follows:

$$K_{v}={\left(\frac{V_{pm}}{V_{pr}}\right)}^{2}$$ (8)

Where *V*_*pm*_ is measured longitudinal wave intensity of tunnel rock (km/s); *V*_*pr*_ is measured longitudinal wave velocity of tunnel rock (km/s).

When the acoustic wave is measured unconditionally, the volumetric joint count Jv of the rock mass can also be used to determine the K_v_ value according to Table 1.

**Table 1:** J_v_ and K_v_ comparison table.

J_v_	<3	3–10	10–20	20–25	>25
K_v_	>0.75	0.75–0.55	0.55–0.35	0.35–0.15	<0.15

The rock mass strength stress ratio in the surrounding rock classification table should be calculated according to the following formula:

1. When there is a measured number of ground stress:
$$S_{m}=\frac{K_{v}f_{r}}{\sigma_{1}}$$ (9)
  Where *S*_*m*_ is rock mass strength stress ratio; *f*_*r*_ is saturated uniaxial compression strength of rock (kPa); *K*_*v*_ is integrity coefficient of rock mass; *σ*_1_ is the maximum principal stress of vertical hole axis (kN/m^2^).
2. When there is no in-situ stress measured data, *σ*_1_ can be determined by the following formula or by displacement back analysis data:
$$\sigma_{1}=\gamma H$$ (10)
  Where *γ* is gravity density of rock mass (kN/m^3^); *H* is thickness of tunnel roof overburden (m).

### 2.5 Calculation of maximum safety span of stope

The reasonable design span is an important factor to ensure the stability of the stope. Therefore, determining the maximum safety span of the stope is also one of the indexes to evaluate the stability of the stope. For the calculation of stope safety span, Barton et al. [22] suggested the following empirical formula to determine the engineering span through field investigation.

$$W=2\cdot ESR\cdot Q^{0.4}$$ (11)

Where W is the maximum safety span of no support system, Q is rock mass quality index, and ESR is the support ratio, which is generally valued according to Table 2. In mining activity, the ESR value is generally selected as 3 according to experience.

**Table 2:** ESR value.

Project Type	ESR
A. Temporary engineering	2~5
B. Permanent mining roadways, water tunnels, auxiliary tunnels, roadways, and chambers	1.6~2
C. Storage chambers, water treatment works Small road and railway tunnels	1.2~1.3
D. Power stations, main roads, and railway tunnels, etc.	0.9~1.1
E. Underground nuclear engineering base, station, public place, factory, or major gas supply tunnel	0.5~0.8

### 2.6 Stope support parameter calculation

The calculation of stope support parameters is also an important content to evaluate the stability of stope and roadway. On the one hand, it provides reasonable suggestions for stope support, on the other hand, it can check the rationality of the supported stope. This comprehensive evaluation method uses empirical formula, national standard recommended method, and Q grading support chart theory to calculate support parameters.

#### 2.6.1 Empirical formula (bolt spacing and length)

According to the existing experience and examples of bolting and shotcrete support, the following empirical formula can be used to determine the bolt parameters for mining projects with a span of less than 10 m:

1. Bolt length L:
$$L=n\left(1.1+\frac{B}{10}\right)$$ (12)
L>2S (13)
  Where B is the roadway span; N is the stability parameter of the surrounding rock (N is 1.0 for the surrounding rock with good stability, N is 1.1 for the surrounding rock with poor stability, N is 1.2 for unstable surrounding rock); S is the joint spacing in the surrounding rock; L is the length of bolt.
  Bolt length takes the larger value according to the above calculation. In the roadway engineering of China, the length of the bolt is generally from 1.5m to 2.0m, whereas 2m to 3m in stope and 2m to 5m in large chamber engineering.
2. Bolt spacing D
D<0.5L (14)
D>3S (15)
  The fracture spacing S is an important factor to determine bolt spacing. In the roadway engineering of China, bolt spacing is generally 0.8m to 1.0m, the longest not more than 1.5m.
3. Bolt diameter d.

Bolt diameter depends mainly on the type of anchor and anchorage force:

$$d\approx \frac{L}{110}$$ (16)

If the bolt length is 2200 mm, the diameter d equals to 20 mm.

However, the above empirical formulas and data ignore the anchoring force of the bolt. The parameters selected by empirical formula, especially the anchorage force, must be tested by theoretical calculation.

#### 2.6.2 China national standard recommended method (anchor spray support)

China national standard “Technical code for the engineering of ground anchorages and shotcrete support” (GB 50086–2015) [21] provides a “Design parameter chart of anchor spray support in slant hole and tunnel”. It demonstrates detailed suggestions for the selection of support parameters of the excavation cavern in combination with surrounding rock grade and excavation span. In the process of selecting support parameters, the type and parameters of bolting and shotcrete support are initially selected according to this chart, and then the mining parameters are modified according to the actual engineering. In addition, when the cavern excavation span is greater than 20m and the height-span ratio H/B is greater than 1.2, the sidewall supporting parameters should be strengthened according to the specific circumstances of the project. When the height-span ratio H/B of the cavern is greater than 2.0, the sidewall support should be strengthened by pre-stressed anchor group support with a length not less than 0.3 times the height of the side wall. The rock pillars between caverns are strengthened according to their thickness or supported by through-type pre-stressed anchor bolts.

#### 2.6.3 Q classification support chart theory

**Fig 7:**
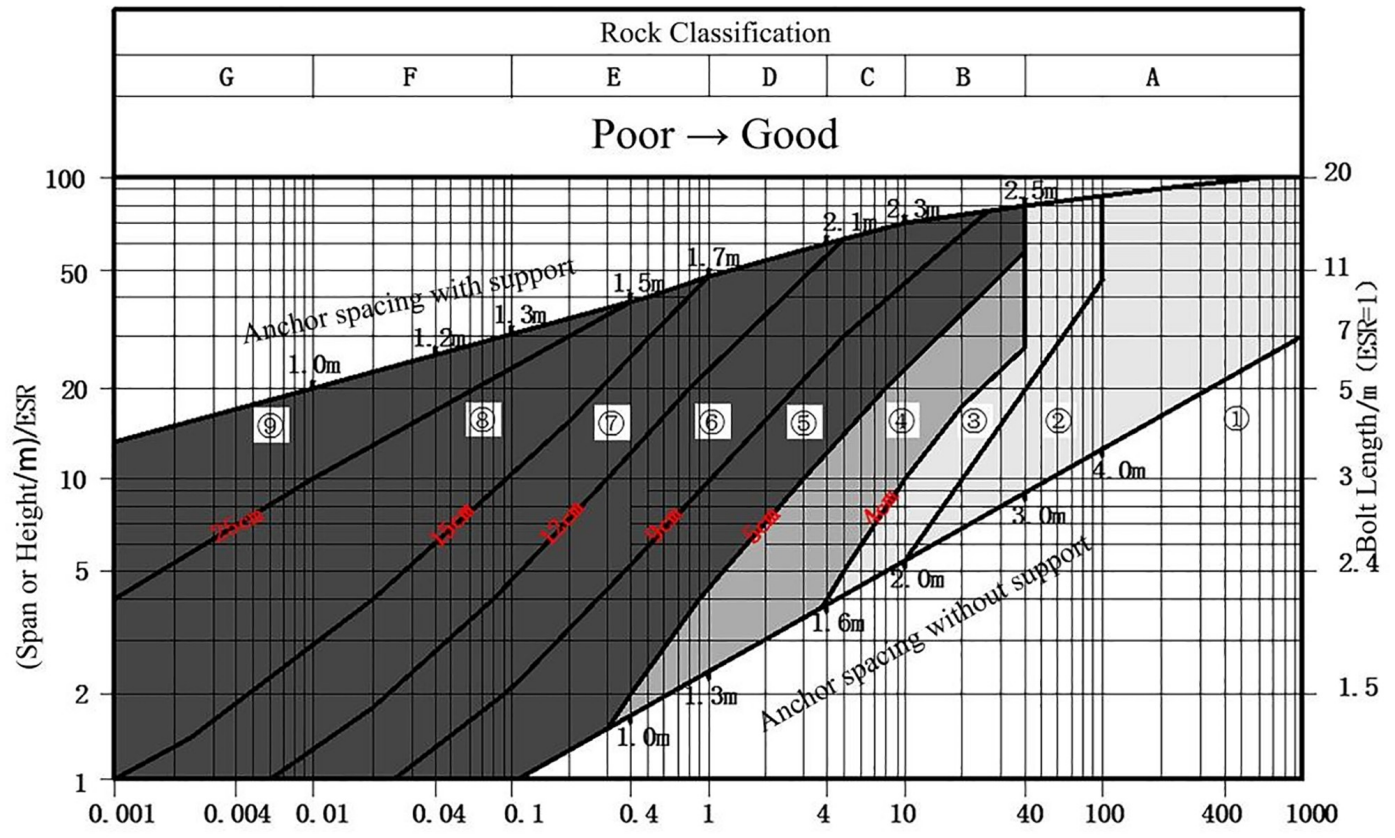
Q system rock classification and support chart. In this figure: ①No support required; ②Point anchor support;③System bolt support; ④System bolt support (plain shotcrete, thickness of 4~5cm); ⑤System bolt and steel fiber shotcrete, thickness 5~9cm; ⑥System bolt and steel fiber shotcrete, thickness of 9~12cm; ⑦System bolt and steel fiber shotcrete, thickness of 12~15cm; ⑧System bolt and steel fiber shotcrete, thickness > 15cm, plus shotcrete reinforced ribs; ⑨Mold concrete lining.

The Barton rock mass quality classification Q system links the rock mass quality with the type of support, and the recommendation of support type is very detailed. According to the Q value, the support design can be divided into 9 categories, as shown in Fig 7 [23]. It shows the latest classification and support charts (based on the statistical results of 2000 tunnels) in the “Norwegian Tunnel and Underground Engineering 2004 Annual Report” published in 2004. It is a series of De-Q value curves from which the required support types can be determined.

The *D*_*e*_ in the Fig 7 is the equivalent size, which is the ratio of the engineering span to the excavation support ratio. The calculation formula is as follows:

$$D_{e}=\frac{\text{Excavation}\;\text{space}\;\text{span}}{\text{ESR}}$$ (17)

### 2.7 Comprehensive evaluation of stope stability

**Fig 8:**
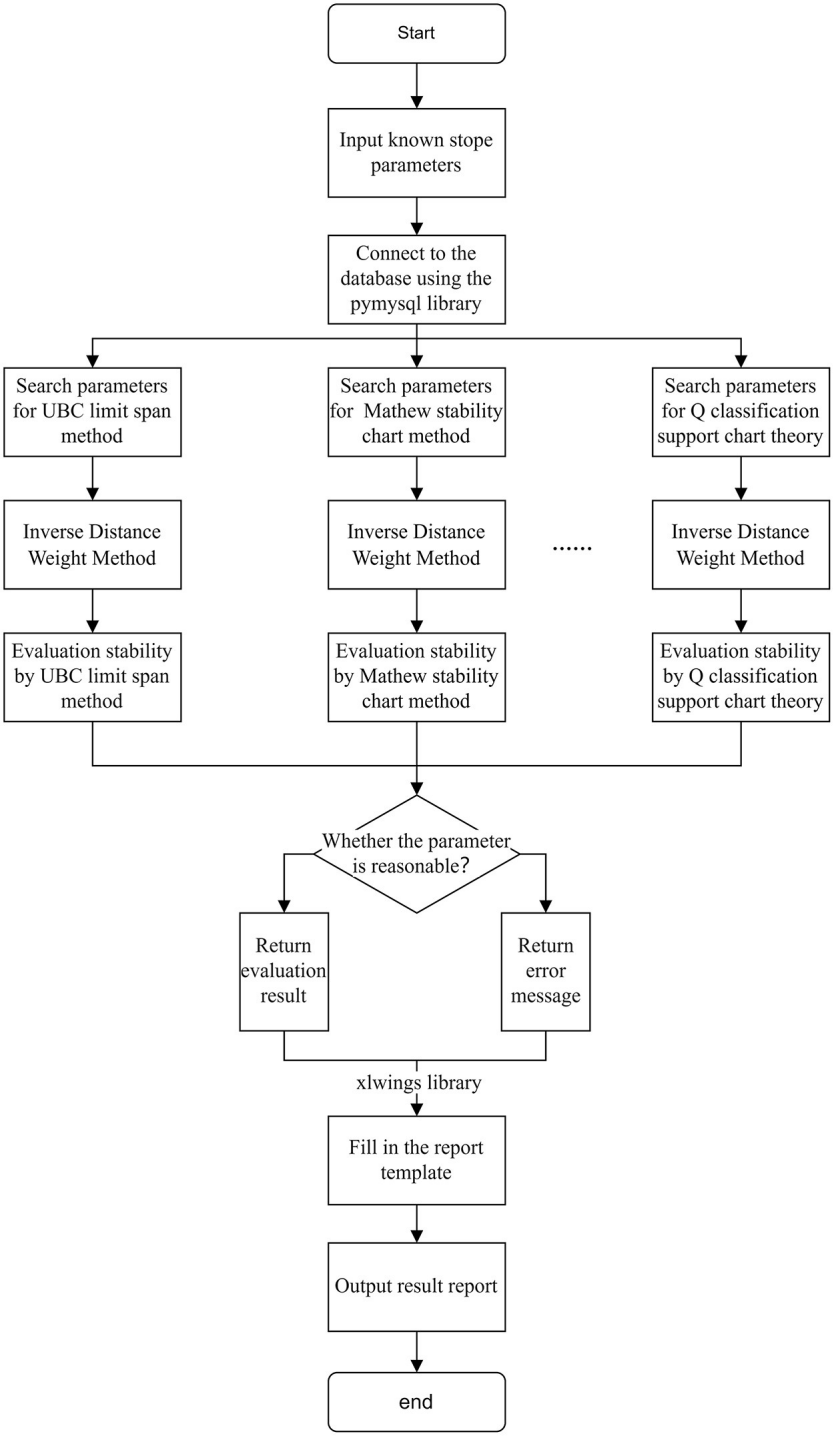
The algorithm flow chart of stope stability comprehensive evaluation system.

The information of rock mechanics parameters investigated is input into a special database. At the same time, in order to better and faster realize the field application, a comprehensive evaluation system of stope stability is compiled by python to realize the comprehensive evaluation of the above various evaluation methods. For the graph curve involved, GetData and Excel are used to get the curve information and fit it. The algorithm flow chart of stope stability comprehensive evaluation system is shown in Fig 8.

In the process of evaluation, the information such as the middle section name, coordinate value and mining parameters of the point that needs to be judged for stability is input to the comprehensive evaluation system of stope stability. The system will find the database to find the parameters of the same middle section according to the input parameters, and then use the inverse distance weight difference method to calculate the parameter information of the input point, so as to evaluate the stability, and finally output the evaluation results of multiple methods to a result report. The generated result report shows the stability evaluation results of four aspects: surrounding rock stability, self-stabilization time of exposed surface, maximum safety span, support parameters and measures.

1. Stability evaluation:
  The Mathews stability chart method and the UBC limit span method are used to evaluate the stability of the input mining parameters at the current evaluation point. The Mathews stability chart method gives three evaluation levels: stability, destruction, and caving. The UBC limit span method gives three evaluation levels: stability, fluctuation, and instability. The two evaluation results confirm each other and will get more accurate stability evaluation.
2. Self-stabilizing time evaluation:
  The self-stabilization time judgment method based on RMR classification obtains its span through the output mining parameters, and then combines the RMR value of the mining point to determine the self-stabilization time of the surrounding rock (in hours) The Chinese national standard recommendation method is based on the self-stabilization time evaluation from the aspects of rock mass structure, rock strength, rock mass acoustic wave, rock mass stress ratio, etc., and outputs the self-stabilization time and failure form of the mining point in the span of 5-10m or below 5m. The two judgment results can confirm each other. If the difference is large, it is necessary to check the rationality of the data in the database and re-evaluate. If the difference is small, it is considered that the judgment result is reasonable, and the smaller value is taken as the self-stabilizing time evaluation value of the evaluation point.
3. Maximum safety span
  The stability evaluation system obtains the Q value of the judgment point in the database, takes the ESR value as 3, and then uses the empirical formula to calculate and output the maximum safety span of the judgment point. In addition, combined with the evaluation results of the UBC limit span method, the rationality of the evaluation is tested. If the two values differ greatly, it is necessary to check the validity of the data. If the difference is not large, a smaller value can be taken as the evaluation result of the maximum safety span.
4. Support parameter calculation
  The empirical formula gives the bolt support suggestion of the judgment point under the input mining parameters, the national standard recommendation method gives the bolt-shotcrete support type and parameter suggestion, and the Q classification support chart theory gives the support parameter design suggestion. The specific implementation of supporting measures has certain subjectivity. The above evaluation methods provide recommended values for the design or optimization of supporting parameters of stope or roadway. The final supporting parameters need to be corrected by mine workers or designers according to the field situation.

## 3 Field application example

### 3.1 Engineering background overview

**Fig 9:**
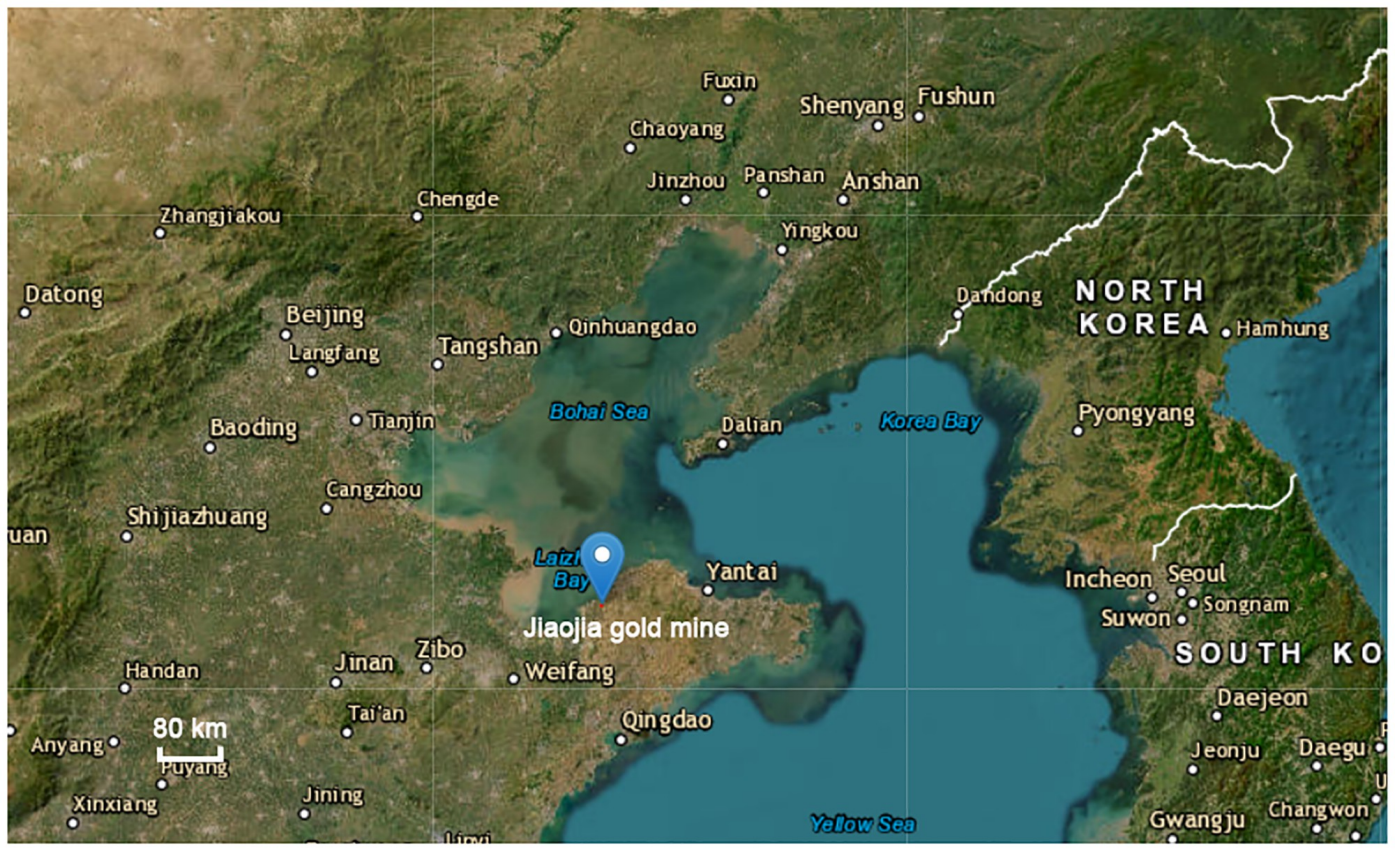
Location of Jiaojia Gold Mine.

Jiaojia Gold Mine is the core enterprise of Shandong Gold Mining Co., Ltd., located in Shandong Province, China (Fig 9). It has three mining areas: Jiaojia, Wangershan and Sizhuang mining area. Among them, the ore body of the Jiaojia mining area occurs in the footwall alteration zone of the main fault. The overall trend of the ore body is NE54°, tendency NW, dip angle of about 27°. The horizontal thickness of the ore body is about 2m to 70m, the thickest is about 70m near the 104 line, and the two wings gradually become thinner to less than 10m. The ore body belongs to gold-bearing pyritization, yellow iron sericite quartzite broken altered rock type, the hanging wall of ore body and the surrounding rock are fault contact relationship, the boundary is obvious; the footwall of the ore body has a gradual transition relationship with the surrounding rock, and there is no obvious boundary. The hanging wall rock of ore body is amphibolite with poor stability, large exposed area or long residence time, which is prone to collapse. The footwall of the ore body is sericitization, silicification and potassic granite, and there are also interlaced fracture joints in it, resulting in local surrounding rock fragmentation. The main mining methods include downward drift cemented filling mining method, upward horizontal drift cemented filling mining method and medium-deep hole double-amplitude sub-section subsequent filling method.

At present, the mining method of Jiaojia Gold Mine mainly has the following problems:

1. The mining method of upward horizontal entry cemented filling has been used for many years in the middle production stage of Jiaojia Gold Mine. The entry section size of most stopes is controlled to 3.5m × 3.5m. The small entry section is beneficial to the safe mining operation, but there are also some problems such as small stope size, small production capacity of single stope, low production efficiency, high production cost and complex construction organization. The production task of Jiaojia Gold Mine in 2021 is 7.6 tons of gold, which is a great challenge. It isn’t easyto successfully complete the heavy production task by continuing to adopt the small-size mining design.
2. The current supporting means of Jiaojia Gold Mine mainly include pipe seam anchor support, anchor net support, anchor net spray support and U-shaped steel arch support. Especially in the stope, the roof joint fissure development, part of the existence of two groups or even multiple groups of joint staggered distribution phenomenon, only through the 1.8 m pipe joint bolt and through the belt to support, often can not meet the requirements of maintaining roof safety.

In summary, it is urgent to count the distribution of joints and fissures in Jiaojia gold mine, classify the stability of ore bodies exposed in different stopes, and optimize the industrial test of upward horizontal approach filling method parameters according to the classification results of ore rock stability, so as to realize efficient mining and successfully complete the production connection task of Jiaojia mining area.

**Fig 10:**
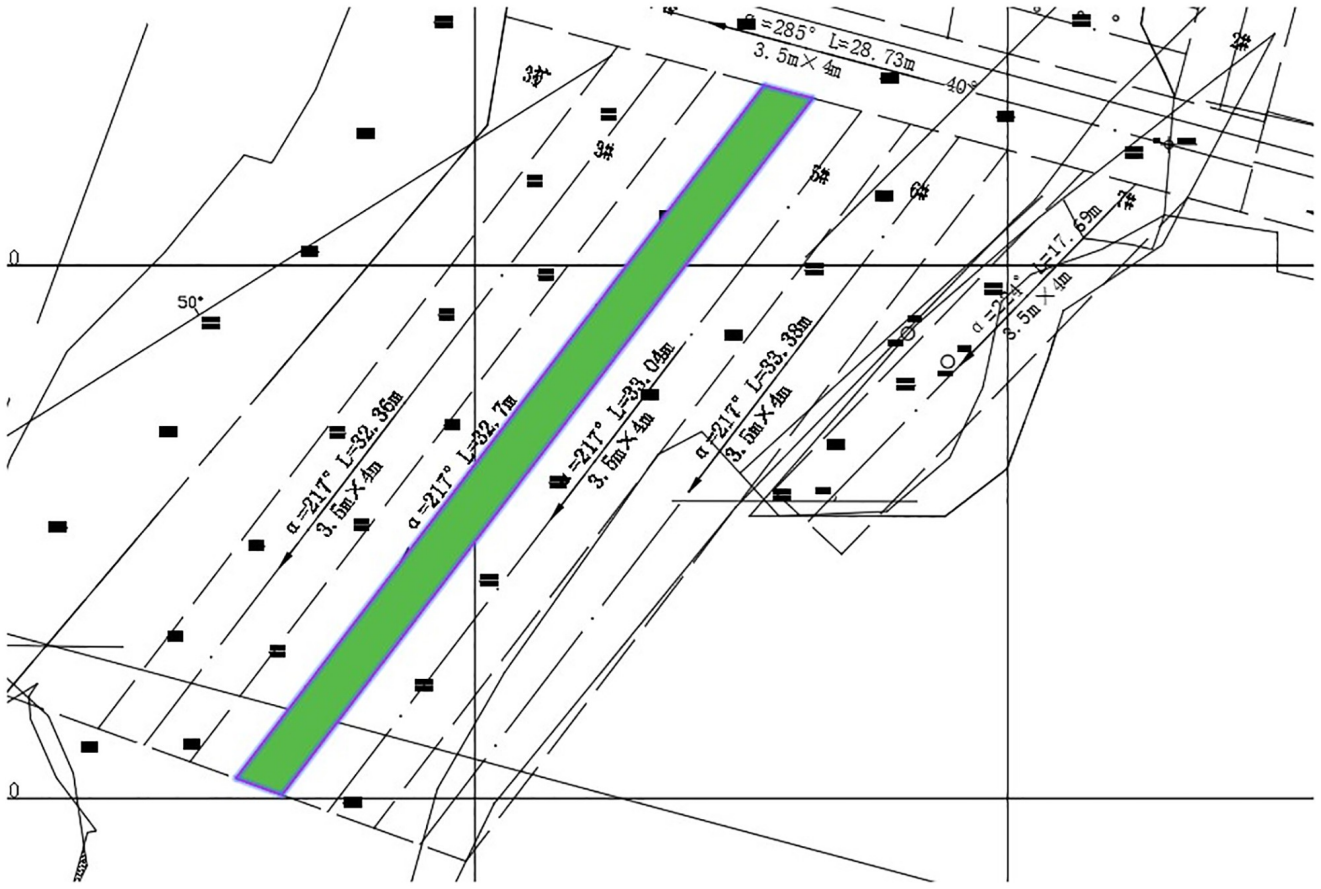
Experimental evaluation of stope design.

This research mainly aims at the rock mechanics data collection of the -570m main production middle section of the mine, and carries on the experimental design stope stability appraisal according to the rock mechanics data and the mine reality, Fig 10 is the mining design drawing of the stope for test evaluation.

### 3.2 Rock mechanics parameters acquisition

#### 3.2.1 Investigation of rock mass structural plane

**Fig 11:**
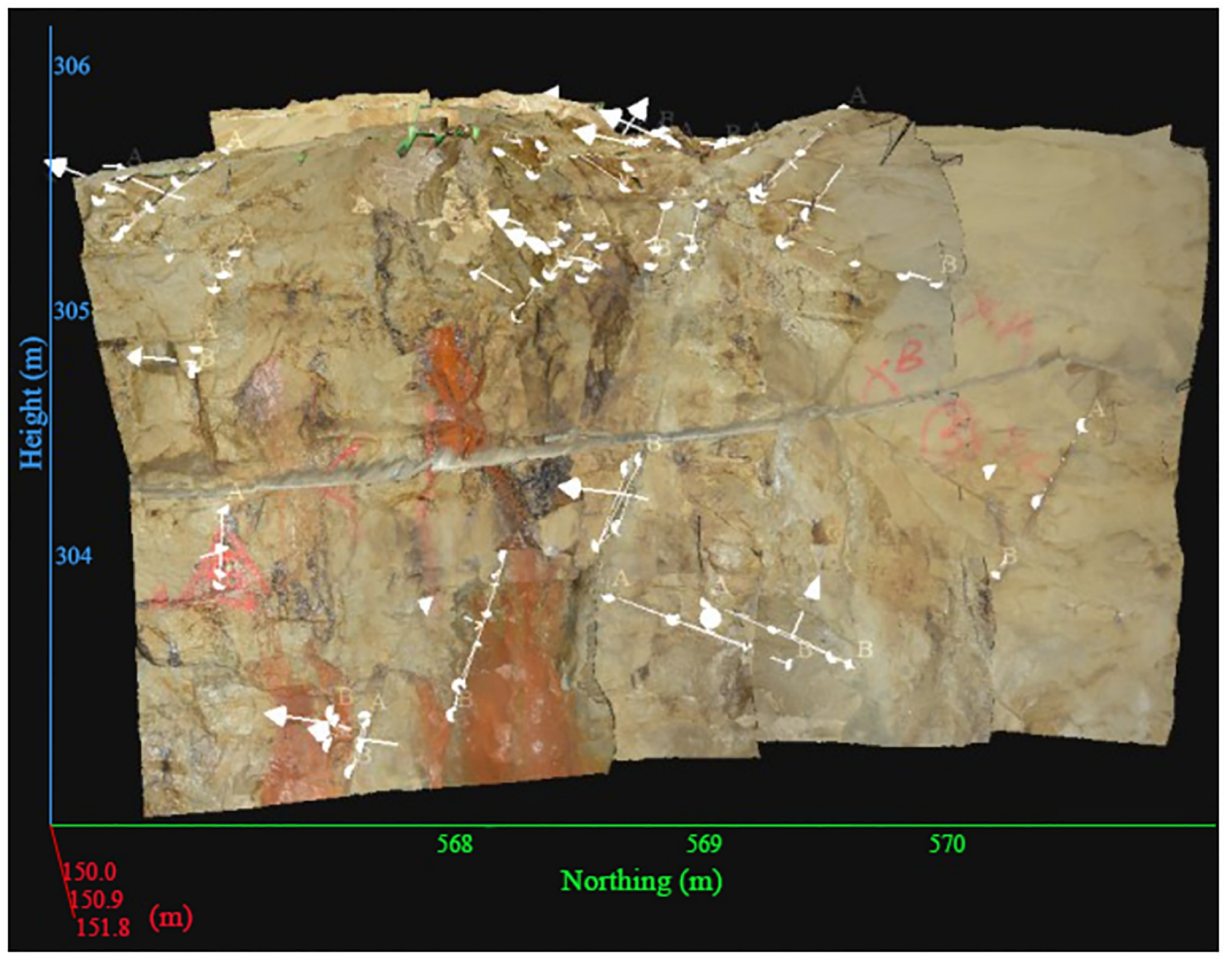
Drawing of rock mass structural plane of measuring point in the -570m level by Sirovision.

**Fig 12:**
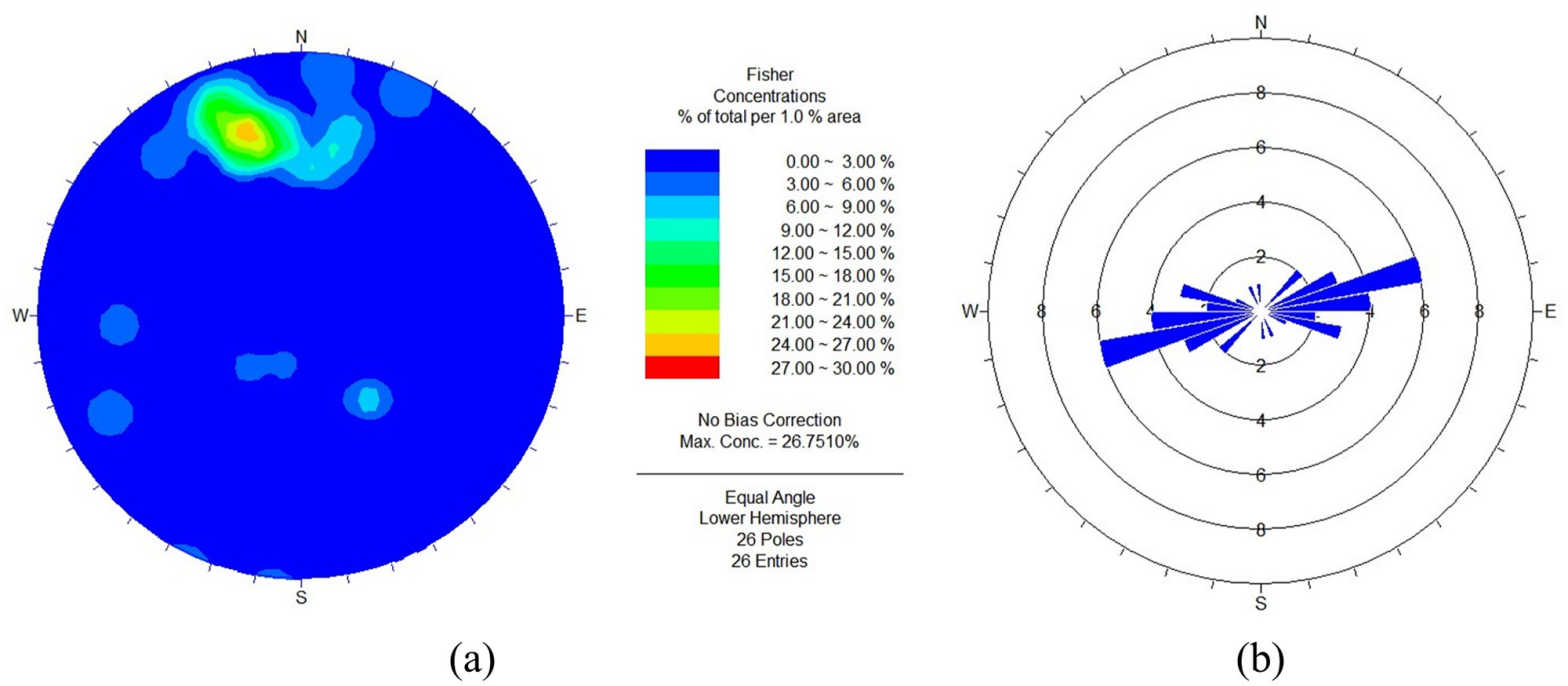
Schmitt diagram and rose diagram of joint in the -570m level (a) Schmitt diagram; (b) rose diagram.

In practical engineering, these small and numerous structural planes are likely to cause large-scale rock engineering problems, that is, rock mass instability. Therefore, in order to explore the failure mechanism of the surrounding rock of the roadway with more developed joints, it is necessary to analyze the characteristics of joint development, such as distribution law, surface characteristics and spatial combination form. In this study, the Sirovision rock mass telemetry and structural analysis system was selected to scan the structural planes of the three-layer and four-layer approach stopes of a sub-lane in the level of the -570 m in the Sizhuang mining area of Jiaojia Gold Mine, and the development of joints and fissures in the area was obtained by software splicing (Fig 11). Combined with Dips6.0 software, the joint measurement results are grouped and counted, and the joint Schmidt diagram and joint rose diagram are drawn, and the results are shown in Fig 12. The information of the -570m level approach structural plane in Sizhuang mining area of Jiaojia Gold Mine is summarized in Table 3. Based on this, Q system and RMR rock mass quality evaluation system are used to classify the rock quality of the surrounding rock in the above area, which provides data basis for the optimization of roadway support and the selection of stope span parameters in this area.

**Table 3:** Structural plane information in the -570m level.

Location	Joint Group Number	Joint Number	Dip (^o^)	Dip Angle (^o^)	Joint Spacing (cm)	Volume Joint Number	Condition of Joint Structural Plane
One lane	2	1	302.7	55.2	16	11.08	The joint fissures of rock mass are relatively developed, and the joints are mostly wavy or rough and irregular. There are fillers between some joints, and the structural plane is relatively wet.
Three-layers							
-570m level		2	89.2	80.3	74		
(9360,9120,-570)							
One lane	2	1	308.5	59.6	22	20.93	
Four-layers							
-570m level		2	90.7	53.0	27		
(9350,9130,-570)							

#### 3.2.2 Rock mechanics experiment

The strength of rock mass has engineering guiding significance for the implementation of ore mining. However, some basic physical and mechanical parameters of rock are needed to calculate the strength of rock mass, so these parameters are the basis of the calculation of the whole stress field. At the same time, the different properties of rock under different conditions have certain reference value for the methods and strategies of field construction.

**Fig 13:**
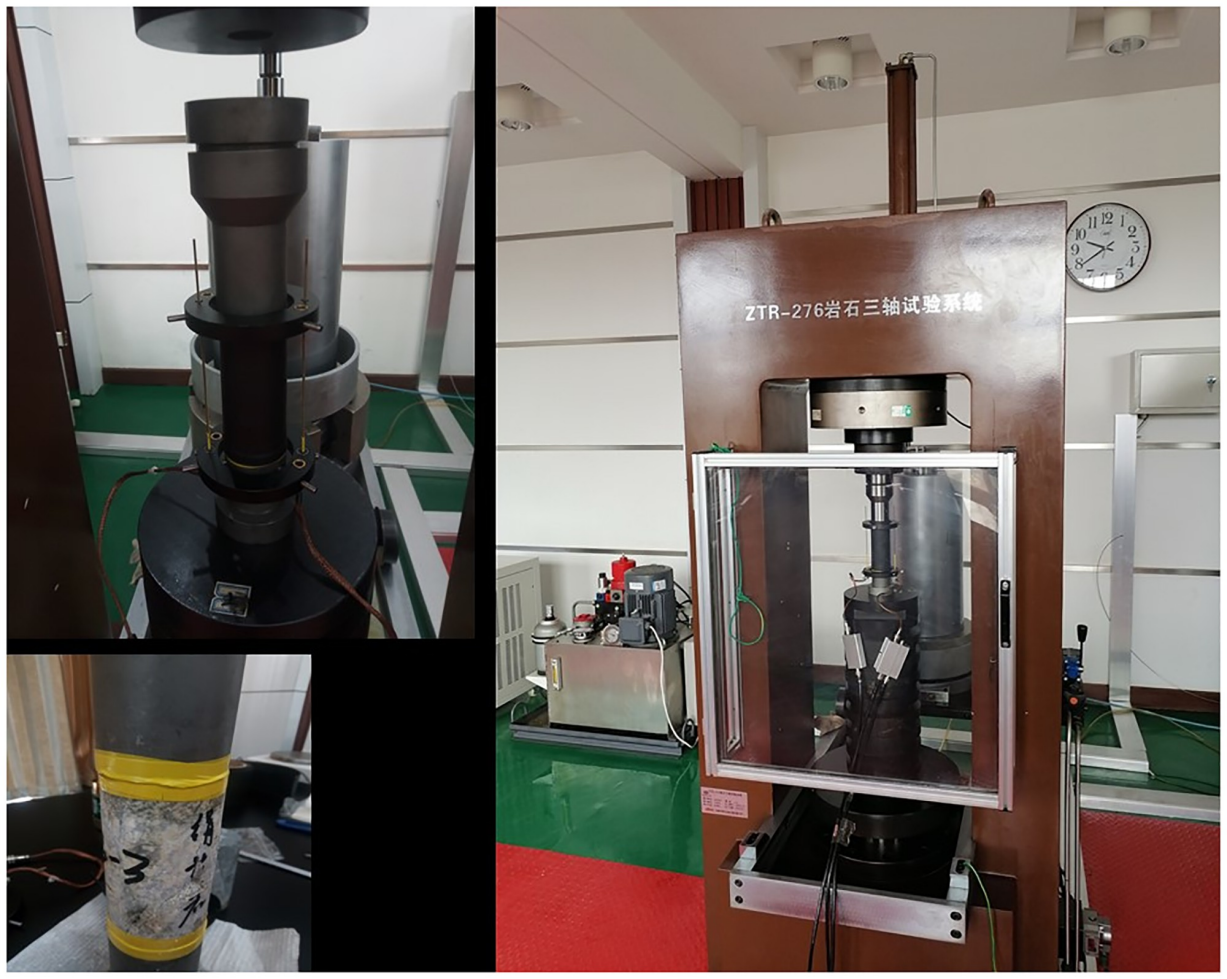
ZTR-276 rock triaxial test system.

The sources of the indoor test samples are mainly roadway sampling and stope sampling. The test rock is mainly granite rock samples. The ZTR-276 rock triaxial test machine is used, and the maximum load is 2000 kN. It includes a loading system, a test system and a control system, as shown in Fig 13. The device has different control modes, such as axial stress control, axial stroke control, axial strain control and cyclic strain control, which can meet the requirements of various experimental designs.

**Fig 14:**
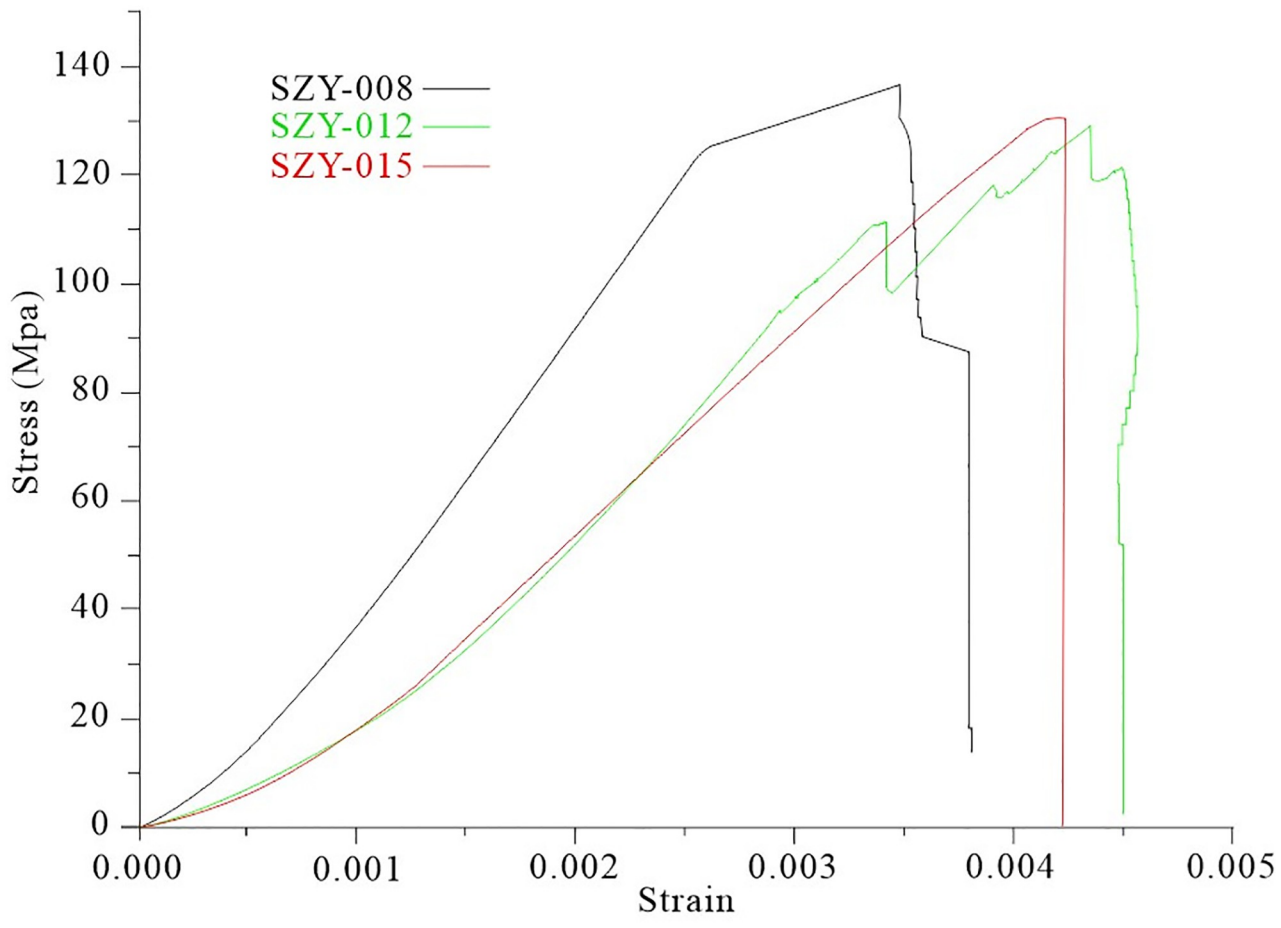
Process curve of rock uniaxial compression in the -570m level of Sizhuang mining area.

We use ZTR-276 rock triaxial test machine to carry out uniaxial loading experiment on the rocks in the -570m middle section of Sizhuang mining area. During the experiment, the loading is controlled by longitudinal strain, and the loading control rate is (1~5) × 10^-6^mm/s. The computer automatically records the original data, such as displacement and load of the experiment, and then draw the stress-strain relationship curve after secondary processing. Fig 14 shows the whole process curve of loading and unloading stress-strain of some samples in the middle section of the -570m.

It can be seen from the Fig 14 that the early curve of rock compressive strength is not smooth, indicating that the rock has joint cracks. During the loading process, the joint cracks weaken the bearing capacity of the rock, and the cement in the rock joint cracks affects the bearing capacity of the rock. There is a small peak curve before the peak strength of the stress-strain curve of the rock sample SZY-008, indicating that there are joints in the rock sample, and its failure strength cannot truly reflect the compressive strength of the rock itself. Therefore, the compressive strength of the rock is 140 MPa. There are many post-peak curves of SZY-012 sample, and it has obvious characteristic curves. After the sample passes the peak stress, its internal structure is destroyed, and the single sample basically maintains the whole shape. In the post-failure stage, the fractures develop rapidly, intersect and combine with each other to form macroscopic fracture surfaces. Since then, the deformation of the rock sample is mainly manifested as the block slip along the macroscopic fracture surface. The bearing capacity of the sample decreases sharply with the increase of deformation, but it does not drop to zero, indicating that the rock sample still has a certain bearing capacity after fracture, but maintains a small value. The strength relative to the final point is called residual strength. With the increase of load until failure, the volume of rock sample increases rather than decreases. This phenomenon is called dilation. The failure of rock sample undergoes a dilation stage, so dilation is often a precursor of rock failure. The results of uniaxial compression test are shown in Table 4.

**Table 4:** Uniaxial compression test results of the -570m level rock.

Location	Lithology	Sample Size (mm)		Compressive Strength (Mpa)	Average Compressive Strength (Mpa)
		Diameter	Height		
One lane	Sericite Rock	49.80	99.80	63.07	133.05
Three-layers					
-570m level		49.60	99.60	136.69	
(9360,9120,-570)		49.70	99.90	129.42	
One lane	Sericite granite	49.60	99.66	144.75	134.10
Four-layers					
-570m level		49.62	99.75	120.27	
(9350,9130,-570)		49.76	100.00	137.28	

### 3.3 Calculation of RMR and Q value

Through the investigation of the surrounding rock at the -570m level in Sizhuang mining area of Jiaojia Gold Mine and the rock mechanics experiment, the rock quality classification of the surrounding rock in the above area is carried out by using Q system and RMR evaluation system, and the classification results are shown in Table 5.

**Table 5:** Rock classification summary.

Location	Q		RMR		
	Value	Description	Value	Grade	Description
One lane	29.42	Good	71	II	Good
Three-layers					
-570m level					
(9360,9120,-570)					
One lane	8.61	General	55	III	General
Four-layers					
-570m level					
(9350,9130,-570)					

### 3.4 Stability evaluation

The investigated rock mechanics parameter information and rock quality classification results will be input into the rock mechanics parameter database associated with the comprehensive evaluation system of the stope. After inputting the location information and mining parameters of the proposed optimization stope or roadway, the system will extract and calculate the corresponding parameters, and evaluate the stability of the area where the point is located. At the same time, it provides suggestions for support optimization and stope span parameter selection.

In this study, the point (9360, 9120, -570) at the three-layer mining and the point (9350, 9130, -570) at the four-layer in the Sizhuang mining area of Jiaojia Gold Mine were selected for evaluation. Among them, the stope in three-layer is 59.84 m long, 5.16m wide and 4.01m high, compared with the stope in four-layer is 31.87m long, 3.5m wide and 3.49m high. Input this information into the stope comprehensive evaluation system, the system will automatically output the results report in the form of excel after calculation.

### 3.5 Stability evaluation results and discussion

For the evaluation points selected in Section 3.4, the main evaluation results of the output report of the stope comprehensive evaluation system are shown in Tables 6 and 7.

**Table 6:** Results of three-layer.

Stope	Jiao Jia Gold Mine, -570m level, One lane, Three layer	
Coordinate	(9360,9120,-570)	
Proposed design parameters	Stope	
	Length: 8m, Width: 5m, Height: 4m	
Rock Classification	RMR	71
	Q	29.42
Stope stability evaluation	UBC limit span method	Scheme in stable area, limit span is 26.95 meters
	Mathews stability chart method	Scheme in stable area
Self-stabilizing time judgment	China national standard recommendation method	When the span is 5m to 10m, rock can be stable for a long time (months to years), only a small part of it falls off
	Self-stabilizing time judgment	The self-stabilizing time without support after mining exposure is estimated to be 42137.59 hours
Maximum safety span without support	Barton stope width empirical formula	The maximum safety span of unsupported area is 23.21m
Support measures and parameters	Empirical formula	Bolt support length 1.76m,
		Bolt spacing less than 0.88m,
		Bolt diameter 16m
	China national standard recommendation method	Shotcrete thickness δ = 50mm
	Q classification support chart theory	No support required

**Table 7:** Results of four-layer.

Stope	Jiao Jia Gold Mine, -570m level, One lane, Four layer	
Coordinate	(9350,9130,-570)	
Proposed design parameters	Stope	
	Length: 8m, Width: 5m, Height: 4m	
Rock Classification	UBC limit span method	55
	Mathews stability chart method	8.61
Stope stability evaluation	China national standard recommendation method	Scheme in stable area, limit span is 16.33 meters
	Self-stabilizing time judgment	Scheme in stable area
Self-stabilizing time judgment	Barton stope width empirical formula	When the span is 5m, the stability time of surrounding rock is very short, about hours to days
	Empirical formula	Self-stabilizing time without support after mining exposure is estimated to be 2454.09 hours
Maximum safety span without support	China national standard recommendation method	Maximum safety span of non-supporting area is 14.20m
Support measures and parameters	Q classification support chart theory	Bolt support length 1.74m,
		Bolt spacing less than 0.87m,
		Bolt diameter 15.42m
	UBC limit span method	1. Shotcrete thickness δ = 80~100mm
		2. Shotcrete thickness δ = 50mm, bolt length 1.5~2.0m, bolt spacing 0.75~1.0m
	Mathews stability chart method	No support required

From Table 6, it can be seen that the surrounding rock quality grade of the three-layer is good. Under the proposed mining parameters, the Mathews chart method and the UBC limit span method are evaluated in the stable area, and the stope is in a stable state. At the same time, under the proposed mining parameters, the national standard recommended method predicts that the surrounding rock of the stope can maintain stability for a long time (months to years), which is also consistent with the 42137.59 hours (about 4 years and 10 months) given by the self-stabilization time judgment. In addition, the maximum safety span of the unsupported area in the Barton stope width empirical formula is 23.21m, and the limit span is 26.95m in the UBC limit span method, which is similar to the Barton stope width empirical formula. Therefore, the maximum safety span of the unsupported area is 23.21m, and the proposed stope structure parameters are within a reasonable range. Finally, the comprehensive evaluation system output different support measures and support parameters from the empirical formula, the national standard recommended method, Q classification support chart theory, which can provide reference for mine site support.

From Table 7, it can be seen that the surrounding rock quality of the four-layer is general. Under the proposed mining parameters, Mathews chart method and UBC limit span method are used to evaluate it in the stable area, and it is concluded that the stope is in a stable state. At the same time, under the proposed mining parameters, the national standard recommended method points out that when the span of the cave is 5m, the stability time of the surrounding rock is very short (about several hours to several days), which is different from the 2454.09 hours (about 3 months) judged by the self-stabilization time method. On the one hand, because the national standard method gives the stability of the 5 m span of the cave, and the evaluation of the stope span is 3.5 m. On the other hand, because the national standard method is located in the standard " Technical Specification for Geotechnical Bolt and Shotcrete Support Engineering " proposed by the Ministry of Housing and Urban-Rural Construction of China. The application in mines still has certain limitations, but no matter which method. Both reflect the poor stability of the surrounding rock of the stope, and the design of reducing the stope span or strengthening the support can be considered. In addition, the maximum safety span of the unsupported project in the Barton stope width empirical formula is 14.20m, and the limit span is 16.33m in the UBC limit span method, which is similar to the Barton stope width empirical formula. Therefore, the maximum safety span of the unsupported project is 14.20m, and the proposed mining parameters are within a reasonable range. Finally, the comprehensive evaluation system proposes the support parameters of different support measures from the three methods of empirical formula, national standard recommendation method and Q classification support chart theory, which needs to be further judged in combination with the actual support method selected on site. It is worth mentioning that due to the small proposed mining parameters of the stope, the theoretical evaluation result of Q grading support chart is no support. However, combined with the evaluation result of self-stabilization time, it can be seen that no support may lead to instability. Whether to support needs to be further judged based on the site situation.

### 3.6 Actual parameters and effect of stope

**Fig 15:**
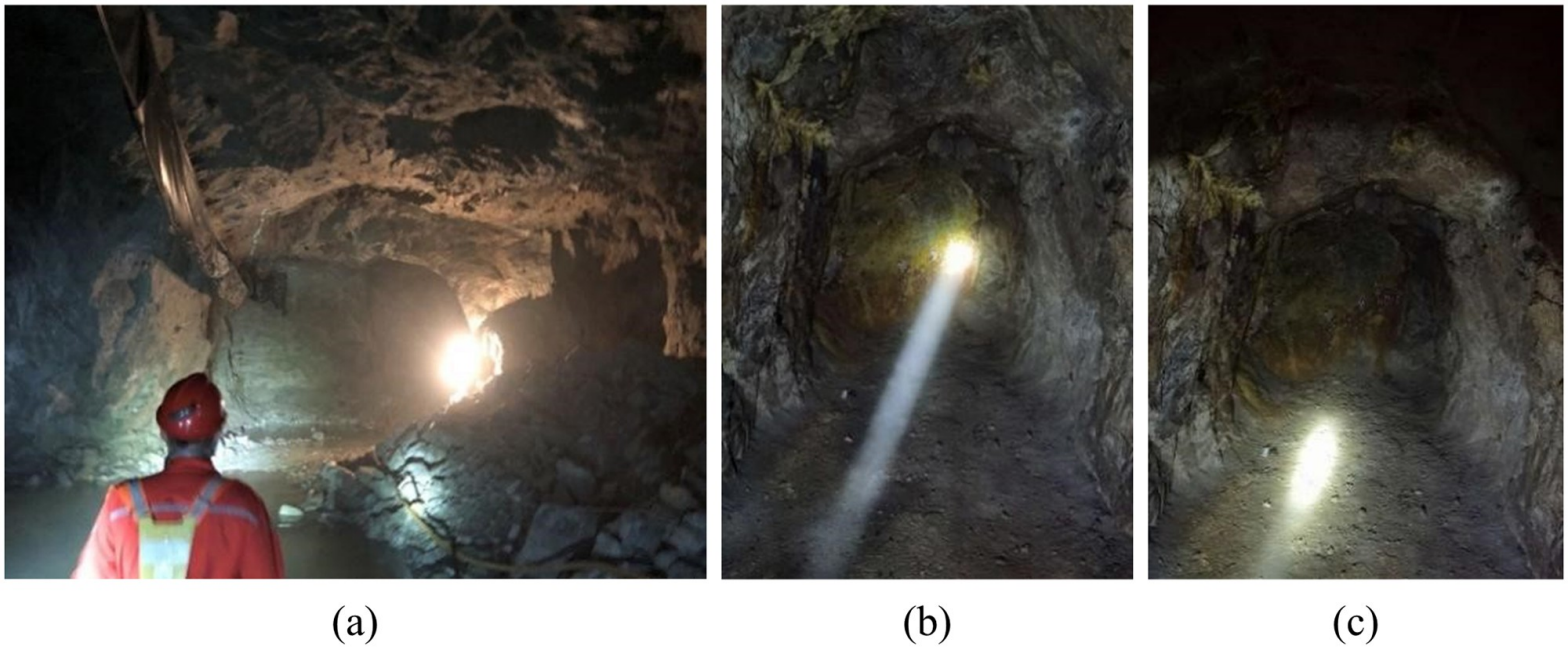
Stope investigation (a)Stope investigation; (b)Work face of three-layer; (c)Work face of four-layer.

In order to verify the accuracy of the system in judging the stability of the stope, the stability of two layers was investigated, as shown in Fig 15. The main production section of Jiaojia Gold Mine has been mined by upward horizontal approach cemented filling mining method for many years. The existing support methods mainly include bolt through belt support, shotcrete support, U-shaped steel arch support, anchor net support and combined support of various situations.

According to the previous rock mechanics investigation, the surrounding rock in three-layer belongs to grade III good rock mass. The final acceptance parameters of the stope are 59.84m long, 5.16m wide and 4.01m high. The support is carried out in the form of 1.8m bolt support, the anchorage spacing is 1m and the row spacing is 3m. The investigation of the ground pressure behavior of the stope for 40 days shows that the stope is relatively stable within 40 days, and there is no obvious ground pressure behavior. According to the field survey results, the stope is in a stable state, the acceptance span is much smaller than the maximum safety span of the unsupported area of 14.20 m, and the self-stability time is longer, which is consistent with the evaluation results of 3.5 sections, and the stope structure parameters are reasonable. At the same time, the stope using bolt support, the parameter selection is better than the recommended parameters in Table 6.

**Fig 16:**
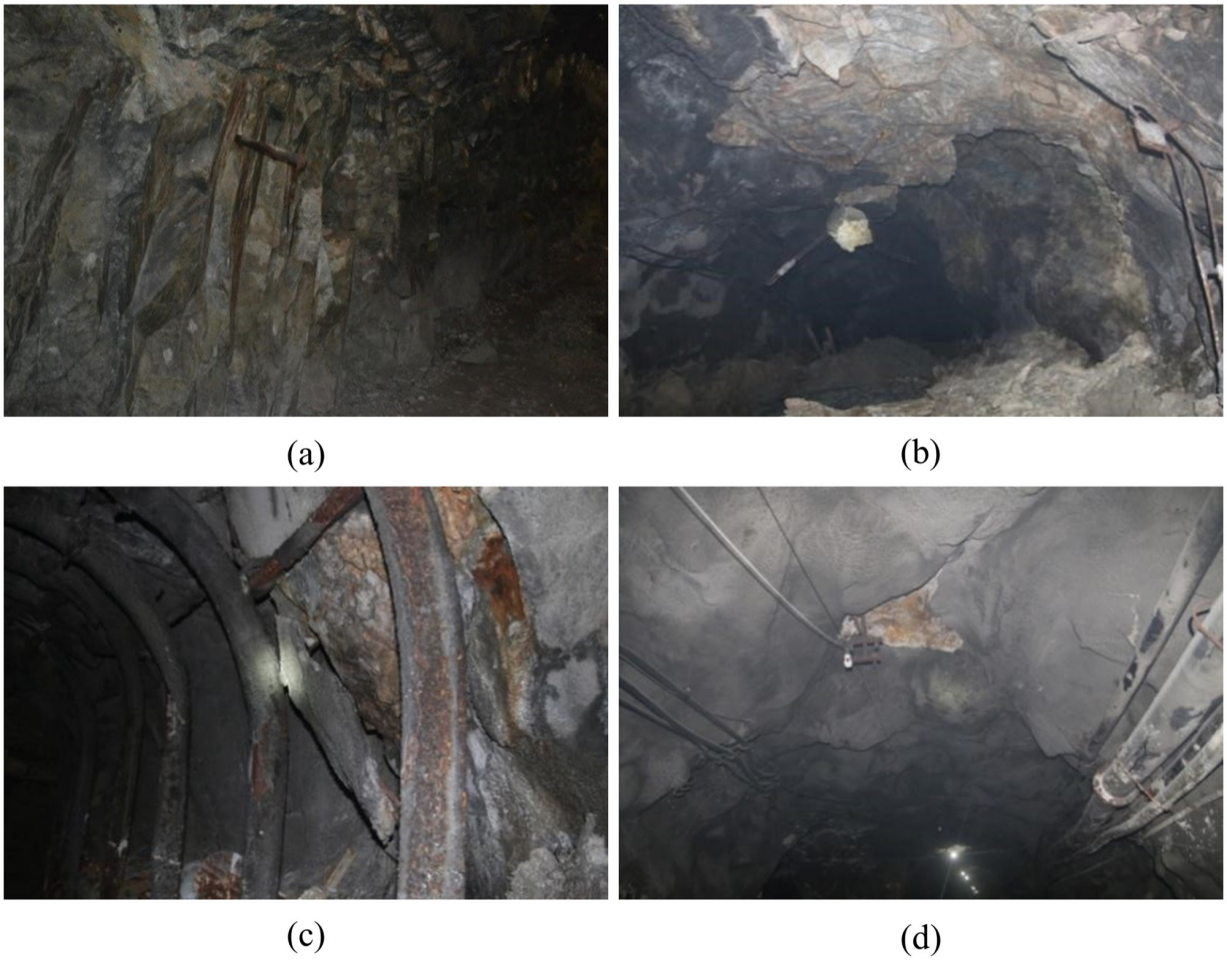
Stope failure in four-layer (a) Bolt support failure; (b) Roof Caving; (c) Cracking of side concrete; (d) Roof concrete caving.

Rock mass in four-layer belongs to II level general rock. The final acceptance parameters of the stope are 31.87m long, 3.5m wide and 3.49m high. The form of bolt-shotcrete combined support is adopted, in which the thickness of shotcrete is 50mm, the length of bolt is 1.8m, the spacing of bolt is 1m, and the row spacing of bolt is 3m. There are many instability phenomena in this stope (Fig 16), in the investigation of the ground pressure behavior of the stope for 14 days, it can be seen that there was a spalling on the 6th day after excavation and a falling block on the 13th day. It can be seen from the survey results that the comprehensive evaluation method gives that the stope is in a stable state under the proposed mining parameters (Table 7), and the acceptance span is much smaller than the maximum safety span of the unsupported area 14.20m, but the actual excavation situation is not optimistic, that is, the situation of spalling and falling occurs on the 6th day after excavation. In fact, this instability situation is also roughly consistent with the judgment result of the self-stability time in the comprehensive evaluation method. It can also be seen that the stability evaluation of the surrounding rock of the stope is multifaceted. The judgment is relatively simple from the span and rock quality classification, while the comprehensive evaluation method adds more evaluation factors. Using multiple evaluation methods to evaluate from multiple dimensions can not only self-test the rationality of each result, but also avoid the limitations of a single evaluation method to a certain extent. In addition, in terms of support, the stope adopts bolt-shotcrete combined support, and its support parameters are consistent with the recommended values in Table 7. However, according to the results of self-stabilization time judgment and field investigation, it needs to be further strengthened.

## 4 Conclusion

In this paper, a comprehensive evaluation method of stope stability is proposed. This method combines Mathews stability chart, limit span method, Chinese national standard recommendation method, self-stabilization time judgment method, Barton stope width empirical formula and Q grading support chart theory. The stope stability is evaluated from four aspects: surrounding rock stability, self-stabilization time, safety span and support parameters, and reasonable suggestions are put forward for the selection of support parameters and measures. The test was carried out in the Sizhuang mining area of Jiaojia Gold Mine. The results show that the comprehensive evaluation method can accurately evaluate the stability of the stope or surrounding rock, and compared with the traditional evaluation method, the evaluation results are more abundant. The sub-evaluation methods can confirm and compare each other, and then provide a more comprehensive and accurate reference for mine design.

However, there are some limitations and imperfections in this study at present: (1) In order to ensure the multidimensional evaluation results, the comprehensive evaluation method uses two non-mine field surrounding rock evaluation methods, namely the Chinese national standard recommendation method mentioned in 2.4 and 2.6 sections, which may interfere with the verification and inspection of the evaluation results. (2) The comprehensive evaluation method pays attention to the multidimensional nature of the evaluation, only simply integrates a variety of surrounding rock stability evaluation methods, the output results are dispersed, and no specific suggestions are put forward for the optimization of structural parameters or support parameters. In view of these shortcomings, in the future, this study is committed to the continuous enrichment and improvement of the comprehensive evaluation method, try to organic integration of various evaluation methods, and combined with the economic benefit analysis of the mine, output more specific and intuitive structural parameters and support parameters optimization value in the results report. In addition, it is necessary to optimize the implementation means of field application, such as the development of evaluation system suitable for mobile communication equipment based on comprehensive evaluation method, which is convenient for mine workers and designers to evaluate stope stability dynamically and quickly.

## Supporting information

S1 Data Structural plane information and uniaxial compression test data. (XLSX)

S2 Data Plot point data for the curve of the charts. This table contains the tracing data and fitting results for the curves in Figs 3, 4, 6, and 7, where the tracing data was generated using GetData.
(XLSX)

S1 Table Evaluation table in GB 50086–2015. Grading table of surrounding rock of tunnel chamber is used for the self-stabilizing time evaluation in Section 2.4.2; type and design parameter table of tunnel and inclined shaft bolting and shotcrete support is used for support parameter assessment in Section 2.6.2.
(DOCX)
