# Supplementary material for: Comprehensive evaluation method of stope stability and its application in deep metal mine
Source: PLoS One. 2023 Mar 17;18(3):e0283205. doi: 10.1371/journal.pone.0283205 (PMC10022794; doi:10.1371/journal.pone.0283205)
Supplement: S1 Table — Grading table of surrounding rock of tunnel chamber is used for the self-stabilizing time evaluation in Section 2.4.2; type and design parameter table of tunnel and inclined shaft bolting and shotcrete support is used for support parameter assessment in Section 2.6.2. (DOCX) [file pone.0283205.s003.docx]

**Grading table of surrounding rock of tunnel chamber in** **GB 50086-2015**

| Grade of surrounding rock | Main engineering geological characteristics | | | | | | | The stable condition |
| --- | --- | --- | --- | --- | --- | --- | --- | --- |
|  | Rock mass structure | Structural influence degree, structural plane development and combination state | Rock strength index | | Acoustic indication of rock mass | | Strength stress ratio of rock mass |  |
|  |  |  | Uniaxial saturation Compressive strength（MPa） | Point load strength（MPa） | Velocity of p-wave in rock mass（km/s） | Rock mass integrity index |  |  |
| Ⅰ | Solid and well interlamellar thick layered structure | Structural influence is slight with occasional minor faults. The structural plane was not developed, only 2 groups to 3 groups, the average spacing was greater than 0.8m, the primary and structural joints were mostly closed, there was no mud filling, not through. The interlayer bond is good, and there is generally no unstable block | >60 | >2.50 | >5 | >0.75 | >4 | When the span is 5m to 10m, the surrounding rock remains stable for a long time and no debris falls |
| Ⅱ | Same class I surrounding rock structure | Characteristics of the same class I surrounding rock | 30-60 | 1.25-2.50 | 3.7-5.2 | >0.75 | >2 | When the span is 5m to 10m, the surrounding rock can maintain stability for a long time (months to years), and only local small pieces fall |
|  | Massive structure and good interlaminar or thick laminar structure | Tectonic influence is heavy, there are a few faults. The structural plane is more developed, generally in 3 groups. The average spacing is 0.4m to 0.8m, mainly primary and structural joints, most of which are closed, occasionally argillaceous filling, poor penetration, and a few weak structural plane. Interlayer bonding is good, occasionally interlayer dislocation and layer opening phenomenon | >60 | >2.50 | 3.7-5.2 | >0.50 | >2 |  |
| Ⅲ | Same class I surrounding rock structure | Characteristics of the same class I surrounding rock | 20-30 | 0.85-1.25 | 3.0-4.5 | >0.75 | >2 | When the span is 5m to 10m, the surrounding rock can maintain stability for more than one month, and there are mainly local blocks and collapses |
|  | The structure is the same as that of the rock mass of grade Ⅱ, and the interlayer combination is good in medium or thick layer structure | The structure is the same as that of the rock mass of grade Ⅱ, and the interlayer combination is good in medium or thick layer structure | 30-60 | 1.25-2.50 | 3.0-4.5 | 0.50-0.75 | >2 |  |
|  | Good interbedded thin layer and soft - hard rock interbedded structure | The structural influence is heavy. Structural plane development, generally in two groups, average spacing 0.2m to 0.4m, mainly structural joints, most of the joint surface closed, little muddy filling. The strata are thin layers or soft and hard rock interbedded mainly with hard rock. The interbedded strata are well integrated, and weak interbedded strata, interbedded dislocation and layer opening are rare | >60（Soft rock ）>，20） | >2.50 | 3.0-4.5 | 0.30-0.50 | >2 |  |
|  | Cataclastic Mosaic structure | The structural influence is heavy. Structural plane development, generally more than 3 groups, average spacing 0.2m to 0.4m, mainly structural joints, most of the joint surface closed, a few are filled with mud, solid interblock occlusion | >60 | >2.50 | 3.0-4.5 | 0.30-0.50 | >2 | When the span is 5m to 10m, the surrounding rock can maintain stability for more than one month, and there are mainly local blocks and collapses |
| Ⅳ | It is the same as the massive structure of the second grade surrounding rock, and the interlayer is well combined with the medium or thick layer structure | It is the same as the massive structure of the second grade surrounding rock, and the interlayer is well combined with the medium or thick layer structure | 10-30 | 0.42-1.25 | 2.0-3.5 | 0.50-0.75 | >1 | When the span is 5m to 10m, the surrounding rock can maintain stability for several days to a month, and the main form of instability is caving or sheet |
|  | Scattered block structure | The structural influence is serious, generally weathering unloading zone. Structural plane development generally consists of 3 groups, with an average spacing of 0.4m to 0.8m, mainly composed of structural joints, unloading and weathered cracks, with good penetration, most of them open and mud trapping. Mud trapping thickness is generally larger than the undulation height of structural plane, and the bite force is weak, forming more unstable blocks | >30 | >1.25 | >2 | >0.15 | >1 | When the span is 5m to 10m, the surrounding rock can maintain stability for several days to a month, and the main form of instability is caving or sheet |
|  | Thin, medium and thick layers and soft and hard rock interbedded structures with poor interbedded bonds | The structural influence is heavy. There are generally more than 3 groups of structural plane development, with an average spacing of 0.2m to 0.4m, mainly structural and weathered joints, most of them are slightly tensioned (0.5mm-1.0mm), and some of them are open (>1.0mm), with mud filling, poor interlayer bonding, most of the mud, interlayer dislocation is obvious | >30（Soft rock），>10） | >1.25 | 2.0-3.5 | 0.20-0.40 | >1 | When the span is 5m to 10m, the surrounding rock can maintain stability for several days to a month, and the main form of instability is caving or sheet |
|  | Cataclastic structure | Structural influence is serious, most of them are fault influence zone or strong Fenghua zone. Structural plane development, generally more than 3 groups, average spacing 0.2m to 0.4m, most of the microtensioned (0.5mm to 1.0mm), part of the open (>1.0mm) has a muddy filling, forming many fragments | >30 | >1.25 | 2.0-3.5 | 0.20-0.40 | >1 |  |
| Ⅴ | Granular structure | Structural influence is serious, most of them are the intersection of fracture zone, full strength weathering zone and fracture zone. The structural and weathered joints are dense, the joint surfaces and their combinations are disorderly, forming a large number of fragments. Most of the blocks are filled with mud, or even stone - soil or earth - stone | - | - | <2.0 | - | - | When the span is 5m to 10m, the surrounding rock stability time is very short, about several hours to several days |

**Type and design parameter table of tunnel and inclined shaft bolting and shotcrete support in GB50086-2015**

|  | Excavation span B, m | | | | | | |
| --- | --- | --- | --- | --- | --- | --- | --- |
| Grade of surrounding rock | B≤5 | 5<B≤10 | 10<B≤15 | 15<B≤20 | 20<B≤25 | 25<B≤30 | 30<B≤35 |
| I | Unsupport | shotcrete thicknessδ=50mm | 1.shotcrete thicknessδ=50-80mm  2.shotcrete thicknessδ=50 mm，bolt length 2.0-2.5m, bolt spacing 1.0-1.5m | shotcrete thicknessδ=100-120mm，bolt length 2.5-3.5，bolt spacing 1.25-1.50 m，If necessary, set up steel mesh | Steel mesh shotcrete thicknessδ=120-150 mm，bolt length 3.0-4.0 m，bolt spacing 1.5-2.0 m | Steel mesh shotcrete thicknessδ=150 mm，bolt length 4.0 and low prestressed anchor bolt length 5.0 m， bolt spacing 1.5-2.0 m | Steel mesh shotcrete thicknessδ=150-200 mm，bolt length 5.0 m and low prestressed anchor bolt length 6.0 m，bolt spacing 1.5-2.0 m |
| Ⅱ | shotcrete thicknessδ=50mm | 1.shotcrete thicknessδ=80-100 mm  2.shotcrete thicknessδ=50mm，bolt length 2.0-2.5 m，bolt spacing 1.0-1.25 m | 1. Steel mesh shotcrete thicknessδ=100-120 mm，Local arrangement of bolt  2. shotcrete thicknessδ=80-100 m，bolt length 2.5-3.5，bolt spacing 1.0-1.5 m，If necessary, set up steel mesh | Steel mesh shotcrete thicknessδ=120-150 mm，bolt length 3.5-4.5 m，bolt spacing 1.5-2.0 m | Steel mesh shotcrete thicknessδ=150-200 mm， bolt length 3.0 m and low prestressed anchor bolt length 4.5 m，bolt spacing1.5-2.0 m | Steel mesh shotcrete thicknessδ=150-200 mm，bolt length 5.0 m and low prestressed anchor bolt length 7.0 m，bolt spacing1.5-2.0 m，prestressed bolt length≥10.0m should be arranged if necessary | Steel mesh shotcrete thicknessδ=180-200 mm，bolt length 6.0 m and low prestressed anchor bolt length 8.0 m，bolt spacing1.5-2.0 m，prestressed bolt length≥10.0m should be arranged if necessary |
| Ⅲ | 1. shotcrete thicknessδ=80-100 mm  2. shotcrete thicknessδ=50 mm，bolt length 1.5-2.0 m，bolt spacing0.75-1.0m | 1．Steel mesh shotcrete thicknessδ=120 mm，Partial arrangement of bolt  2. Steel mesh shotcrete thicknessδ=80-100 mm，bolt length 2.5-3.5m，bolt spacing1.0-1.5 | Steel mesh shotcrete thicknessδ=100-150 mm，bolt length 3.5-4.5m，bolt spacing1.5-2.0，local reinforcement | Steel mesh or steel fiber shotcrete thicknessδ=150-200 mm，bolt length 3.5-5.0m，bolt spacing1.5-2.0m，local reinforcement | Steel mesh or steel fiber shotcrete thicknessδ=150-200 mm，bolt length 4.0锚杆and low prestressed anchor bolt length 6.0m，bolt spacing1.5m，prestressed bolt length≥10.0m should be arranged if necessary | Steel mesh or steel fiber shotcrete thicknessδ=180-250 mm，bolt length 6.0锚杆and low prestressed anchor bolt length 8.0m，bolt spacing1.5m，prestressed bolt length≥15.0m should be arranged if necessary | Steel mesh or steel fiber shotcrete thicknessδ=200-250 mm，bolt length 6.0 mand low prestressed anchor bolt length 9.0m，bolt spacing1.2-1.5m，prestressed bolt length≥15.0m should be arranged if necessary |
| Ⅳ | Steel mesh shotcrete thicknessδ=80-100 mm，bolt length 1.5-2.5m，bolt spacing1.0-1.25m | Steel mesh shotcrete thicknessδ=120-150 mm,low prestressed anchor bolt length L=2.0-3.0mbolt spacing1.0-1.25m，Erect inverts and implement secondary support if necessary | Steel mesh or steel fiber shotcrete thicknessδ=200 mm， low prestressed anchor bolt length 4.0-5.0，bolt spacing1.0-1.25m，Local steel arch or grille arch, invert or secondary support if necessary | - | - | - | - |
| V | Steel mesh shotcrete thicknessδ=150 mm，bolt length 1.5-2.5m，bolt spacing0.75-1.25m，erect invert and implement secondary support | Steel mesh shotcrete thicknessδ=200 mm，and low prestressed anchor bolt length L=2.5-3.5m，bolt spacing0.75-1.0m，local steel arch or grid arch, inverted arch or secondary support |  |  |  |  |  |
